# WAND: A multi-modal dataset integrating advanced MRI, MEG, and TMS for multi-scale brain analysis

**DOI:** 10.1038/s41597-024-04154-7

**Published:** 2025-02-06

**Authors:** Carolyn B. McNabb, Ian D. Driver, Vanessa Hyde, Garin Hughes, Hannah L. Chandler, Hannah Thomas, Christopher Allen, Eirini Messaritaki, Carl J. Hodgetts, Craig Hedge, Maria Engel, Sophie F. Standen, Emma L. Morgan, Elena Stylianopoulou, Svetla Manolova, Lucie Reed, Matthew Ploszajski, Mark Drakesmith, Michael Germuska, Alexander D. Shaw, Lars Mueller, Holly Rossiter, Christopher W. Davies-Jenkins, Tom Lancaster, C. John Evans, David Owen, Gavin Perry, Slawomir Kusmia, Emily Lambe, Adam M. Partridge, Allison Cooper, Peter Hobden, Hanzhang Lu, Kim S. Graham, Andrew D. Lawrence, Richard G. Wise, James T. R. Walters, Petroc Sumner, Krish D. Singh, Derek K. Jones

**Affiliations:** 1 Cardiff University Brain Research Imaging Centre, School of Psychology, Cardiff University, Cardiff, UK; 2 Department of Psychology, Durham University, Durham, UK; 3 Department of Psychology, Royal Holloway, University of London, Egham, UK; 4 School of Psychology, Aston University, Birmingham, UK; 5 Department of Radiology, University of California Davis Medical Center, Sacramento, California USA; 6 Washington Singer Laboratories, University of Exeter, Exeter, UK; 7 Leeds Institute of Cardiovascular and Metabolic Medicine, University of Leeds, Leeds, UK; 8 The Russell H. Morgan Department of Radiology and Radiological Science, Johns Hopkins University School of Medicine, Baltimore, Maryland USA; 9 F.M. Kirby Research Center for Functional Brain Imaging, Kennedy Kreiger Institute, Baltimore, Maryland USA; 10 Department of Psychology, University of Bath, Bath, UK; 11 IBM Polska Sp. z o. o., Department of Content Design, Cracow, Poland; 12 University of Sheffield, Research Services, New Spring House, 231 Glossop Road, Sheffield, S10 2GW UK; 13 School of Philosophy, Psychology and Language Sciences, Dugald Stewart Building, University of Edinburgh, 3 Charles Street, Edinburgh, EH8 9AD UK; 14 Department of Neurosciences, Imaging, and Clinical Sciences, ‘G. D’Annunzio’ University of Chieti-Pescara, Chieti, Italy; 15 Institute for Advanced Biomedical Technologies, ‘G. D’Annunzio’ University of Chieti-Pescara, Chieti, Italy; 16 School of Medicine, Centre for Neuropsychiatric Genetics and Genomics, Cardiff University, Cardiff, UK

**Keywords:** Neuroscience, Human behaviour, Cognitive neuroscience

## Abstract

This paper introduces the Welsh Advanced Neuroimaging Database (WAND), a multi-scale, multi-modal imaging dataset comprising *in vivo* brain data from 170 healthy volunteers (aged 18–63 years), including 3 Tesla (3 T) magnetic resonance imaging (MRI) with ultra-strong (300 mT/m) magnetic field gradients, structural and functional MRI and nuclear magnetic resonance spectroscopy at 3 T and 7 T, magnetoencephalography (MEG), and transcranial magnetic stimulation (TMS), together with trait questionnaire and cognitive data. Data are organised using the Brain Imaging Data Structure (BIDS). In addition to raw data, we provide brain-extracted T1-weighted images, and quality reports for diffusion, T1- and T2-weighted structural data, and blood-oxygen level dependent functional tasks. Reasons for participant exclusion are also included. Data are available for download through our GIN repository, a data access management system designed to reduce storage requirements. Users can interact with and retrieve data as needed, without downloading the complete dataset. Given the depth of neuroimaging phenotyping, leveraging ultra-high-gradient, high-field MRI, MEG and TMS, this dataset will facilitate multi-scale and multi-modal investigations of the healthy human brain.

## Background & Summary

The development of neuroimaging techniques, such as magnetic resonance imaging (MRI), magnetoencephalography (MEG) and transcranial magnetic stimulation (TMS), that manipulate or exploit the magnetic properties of tissue, has revolutionised the field of neuroscience and our understanding of the living human brain^1–3^. Advances in magnetic resonance technology have resulted in the development of ultra-high static magnetic fields^4^ and ultra-strong magnetic gradients^5^ for human MRI, that facilitate imaging at high spatial resolutions, or with strong diffusion weightings, respectively. These techniques allow researchers to non-invasively probe the macro and micro-structural properties of the human brain^5^, improve spatial resolution and contrast of tissue^6–8^, improve the signal to noise ratio and measurement precision of metabolite spectra^9^ and allow for finer localisation of blood oxygen changes in blood oxygen level dependent (BOLD) functional MRI^10–12^. Combined with the millisecond temporal resolution of MEG and the ability to manipulate neuronal activity using TMS, these cutting-edge technologies can help to describe the complex coupling between brain structure, function, and behaviour, essential to the progression of neuroscientific research.

Despite the value of advanced multi-modal imaging, limited access to resources or funding often restricts the scope of individual studies to one or two imaging modalities, to a small number of participants, or to a limited number of well-resourced imaging centres, limiting the interpretability of findings. Thanks to large-scale collaborations and recent data sharing initiatives, however, large-scale multi-modal datasets, such as the Human Connectome Project (HCP)^13^, Adolescent Brain Cognitive Development (ABCD) study^14^, Cambridge Centre for Ageing and Neuroscience (Cam-CAN) data repository^15^, developing Chinese Color Nest Project^16^, the I See Your Brains (ISYB) dataset^17^, The Latin American Brain Health Institute (BrainLat) project^18^, UK Biobank, Chinese Human Connectome Project, and Multimodal Imaging and Connectome Analysis dataset for Microstructure-Informed Connectomics (MICA-MICs)^19^ are now publicly available. Each of these datasets provides unique data combinations and opportunities for investigating complex brain-behaviour relationships as well as multi-scale human brain mapping^20^,^21^.

However, in spite of these large-scale efforts, our understanding of how biological processes at the micro-scale (e.g., cellular structure or synaptic function) relate to metabolic and functional processes at the macro-scale (functional network or whole brain) remains limited. The Welsh Advanced Neuroimaging Database (WAND) has been especially designed to equip researchers with the data necessary to begin tackling this multi-scale problem.

WAND^22^ comprises non-invasive *in vivo* brain data from 170 healthy adult volunteers and is the first publicly available neuroimaging dataset to combine cutting-edge 3 T MRI with ultra-strong (300 mT/m) magnetic field gradients (especially designed for evaluating tissue microstructure), 7 T and 3 T MRI and nuclear magnetic resonance spectroscopy (MRS), MEG, and TMS, together with demographic, cognitive, and trait questionnaire data within individual participants.

The unique and rich combination of data in WAND^22^, acquired using state-of-the-art technology, and targeting specific features of brain structure and function, allows for investigation of brain-behaviour relationships at multiple spatial and temporal scales. The dataset consists of macro- and micro-structural, functional, perfusion, metabolic, and behavioural measurements, that can be combined to address a multitude of research questions related to multi-scale coupling in the human brain.

Integrating data from multiple complementary imaging modalities will provide numerous benefits over single modality analyses. Within the functional imaging domain, the benefits of high spatial resolution from fMRI and high temporal resolution from MEG can be combined to better pinpoint neural connectivity among specific brain regions^23,24^. Moreover, integrating cerebral blood flow data enables researchers to model the inter-individual variability in blood flow that could impact neurovascular coupling^25,26^. Microstructural, neurointerventional and neurochemical data from diffusion MRI, myelin imaging and MR spectroscopy could also be incorporated, to validate or strengthen predictive models of brain function, improving estimations for techniques such as dynamic causal modelling^27^. Combining these data could also be useful for investigating theories of conduction delay^28^, structure-function coupling^29^, and neurochemical associations of functional brain signatures^30^, as well as addressing technical challenges, such as image quality transfer^31^.

WAND^22^ is free to access and provides flexible download options. Data are organised using the Brain Imaging Data Structure (BIDS)^32^, and accompanied by a well-documented git repository, as well as quality reports for several modalities. Full descriptions of acquisition protocols and data for each imaging and research modality are provided here.

## Methods

### Participants

**Fig. 1:**
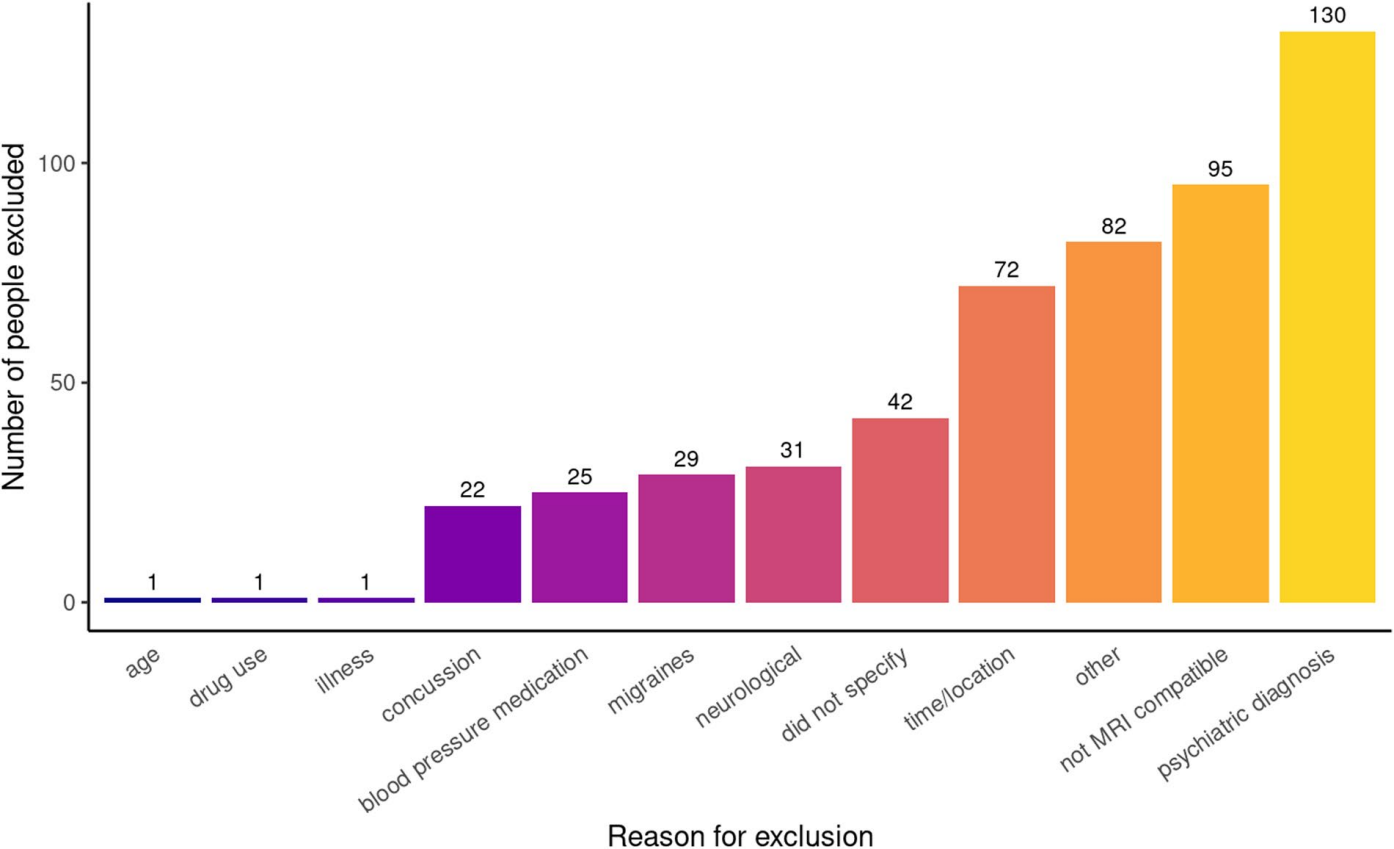
Reasons for exclusion of individuals from the Welsh Advanced Neuroimaging Database.

**Fig. 2:**
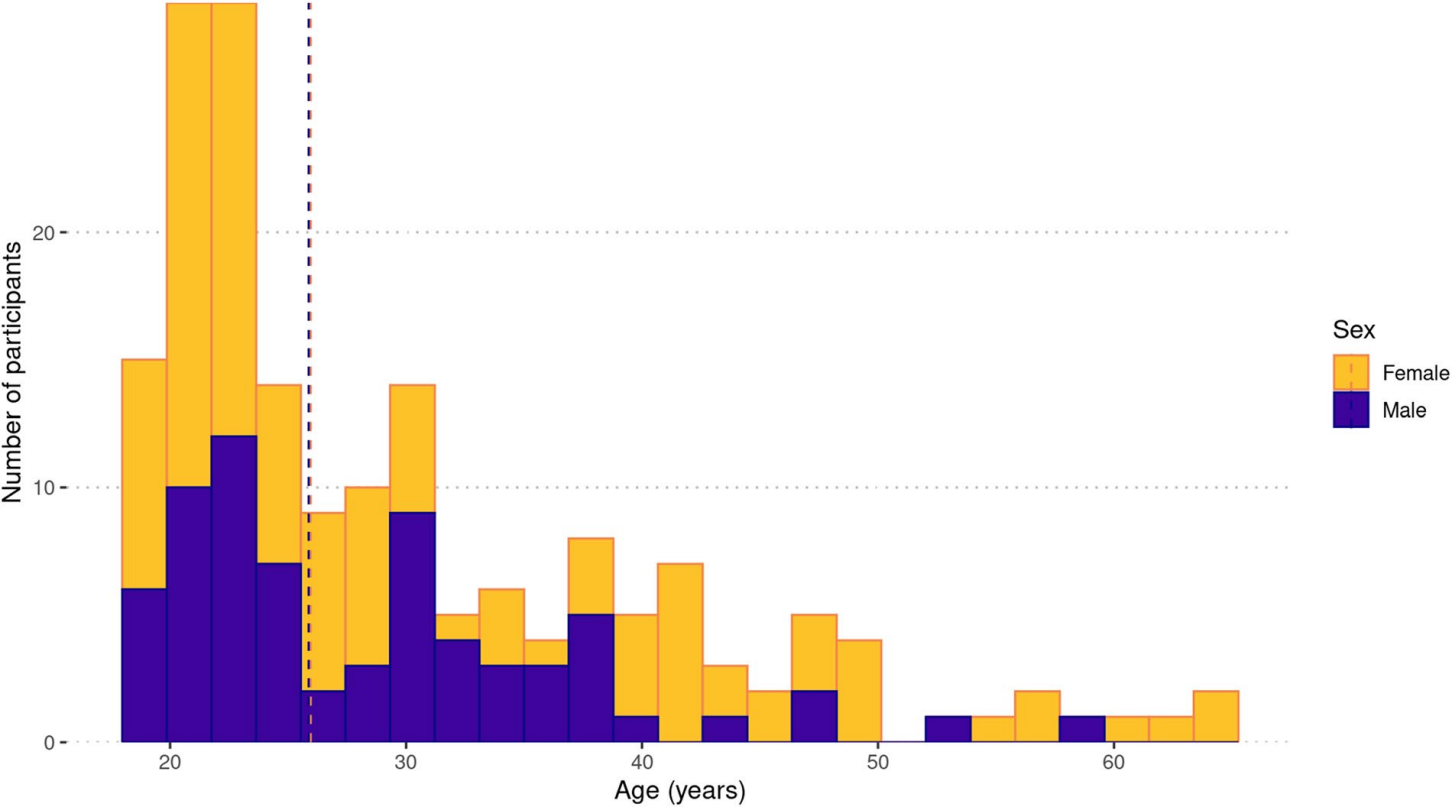
Age distribution of WAND participants. Females are shown in yellow; males are shown in purple. Dashed lines represent the median age for each sex.

**Fig. 3:**
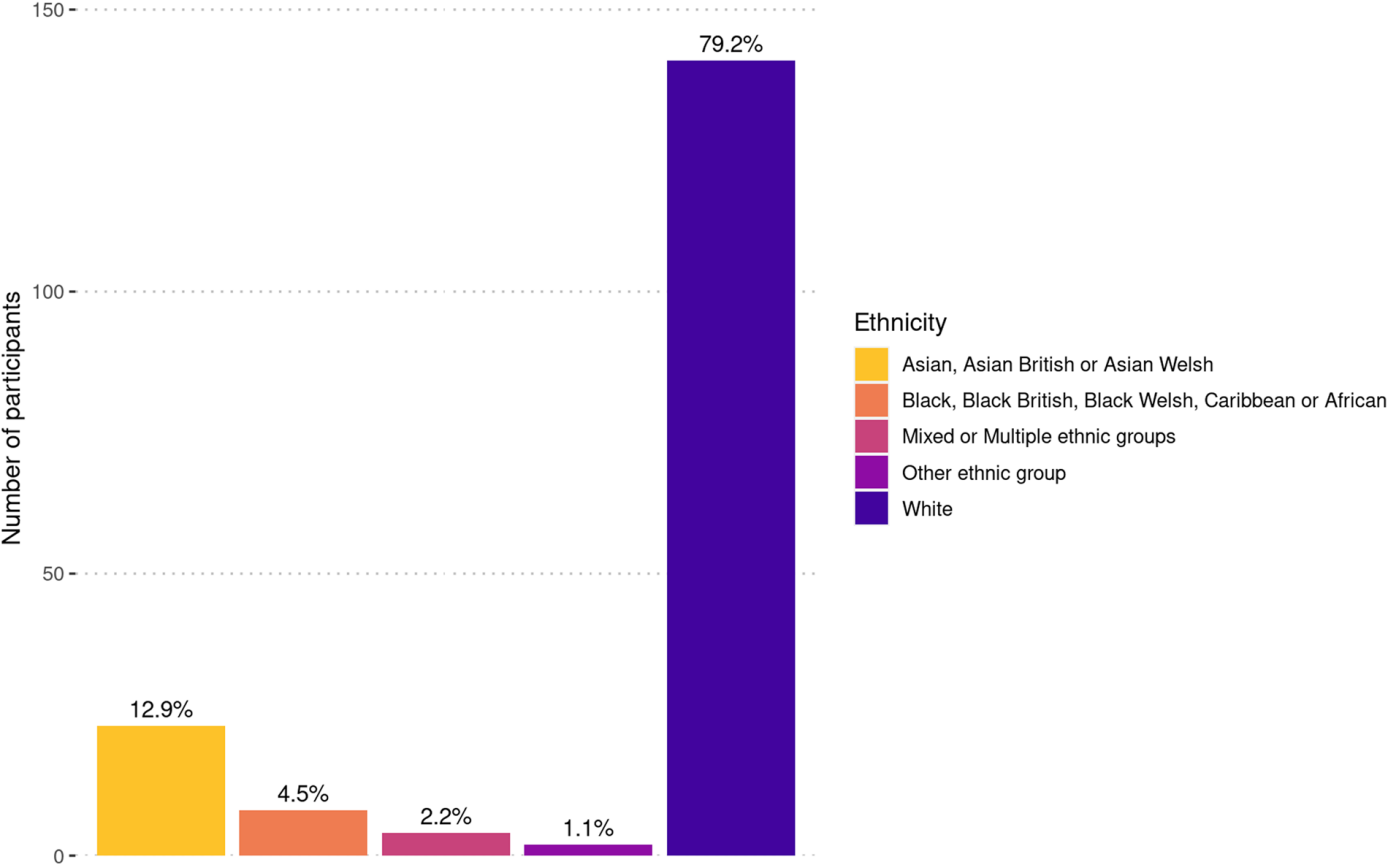
Summary of self-reported ethnicity data across WAND participants. Data were categorised using ethnic group classifications from the 2021 UK Census, as reported by the Office for National Statistics (https://www.ons.gov.uk/census/census2021dictionary/variablesbytopic/ethnicgroupnationalidentitylanguageandreligionvariablescensus2021/ethnicgroup/classifications).

Healthy volunteers were recruited from Cardiff, Wales, United Kingdom, and surrounding areas between February 2019 and May 2023. The study was advertised through the Cardiff University community page (Yammer) and emails, as well as through Cardiff University’s Experimental Management System (EMS), in person during university orientation week and via posters placed around campus. Community members were recruited through Healthwise Wales and via word of mouth. A total of 717 individuals were screened for eligibility, of whom 178 (108 female) were enrolled, and 170 had at least one scan. Figure 1 and Table S1 provide a summary of the primary reasons for exclusion. All participants were between 18 and 63 (median 25) years of age (see Fig. 2 for age distributions of male and female participants). Ethnicity was self-reported by study participants and later categorised using classifications from the 2021 UK Census, as reported by the Office for National Statistics (https://www.ons.gov.uk/census/census2021dictionary/variablesbytopic/ethnicgroupnationalidentitylanguageandreligionvariablescensus2021/ethnicgroup/classifications). Based on results from the 2021 Census^33^, the ethnic distribution of the WAND sample was consistent with the population of Wales (where the data were collected), though with higher proportions of ethnic minorities (see Fig. 3): 79.2% White (vs 93.8% in 2021 Census); 12.9% Asian (vs 2.9% in 2021 Census); 4.5% Black (vs 0.9% in 2021 Census); 2.2% Mixed or multiple ethnic groups (vs 1.6% in 2021 Census); and 1.1% Other (vs 0.9% in 2021 Census).

**Fig. 4:**
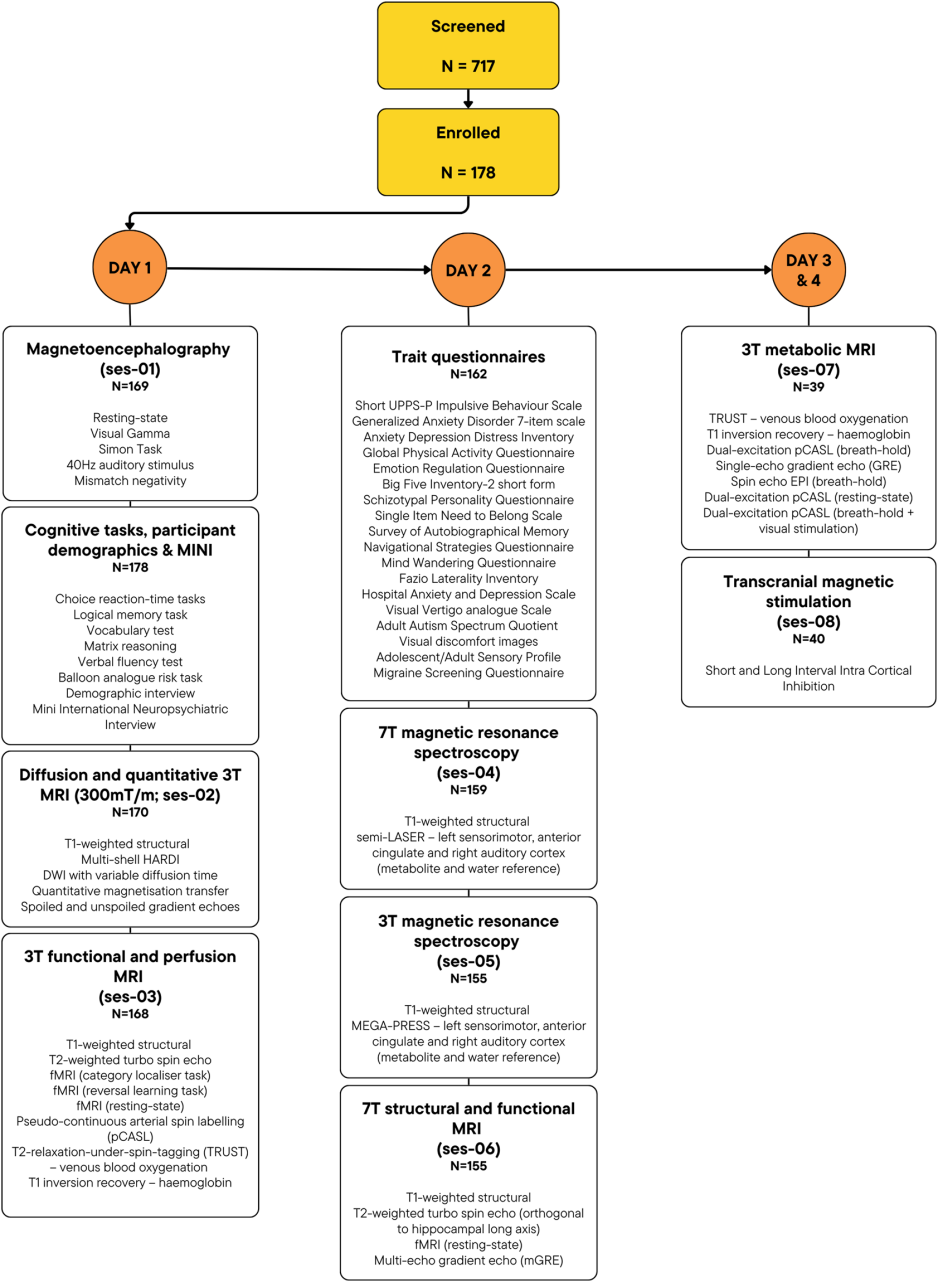
Recruitment and participant flow through the Welsh Advanced Neuroimaging Database. Numbers presented are the total number of individuals with any data for that imaging session (ses). Median number of days between testing was 2 (day 1 to day 2), 8 (day 2 to day 3) and 6 (day 3 to day 4). MINI: Mini International Neuropsychiatric Interview; HARDI: high angular resolution diffusion-weighted imaging; DWI: diffusion-weighted imaging; fMRI: functional magnetic resonance imaging; pCASL: Pseudo-continuous arterial spin labelling; TRUST: T2-relaxation-under-spin-tagging.

Participants were invited to the Cardiff University Brain Research Imaging Centre (CUBRIC), to take part in up to 8 neuroimaging sessions plus additional cognitive testing. The flow of participants is shown in Fig. 4. Each participant underwent two days of testing, with a sub-sample of 40 participants returning for two additional days (TMS and 3 T metabolic scans). All participants were required to be between 18 and 65 years of age (with a later adjustment to between 18 and 45 years of age due to recruitment challenges) and meet MR safety requirements for scanning. Exclusion criteria included diagnosis of any heart or breathing problem; high blood pressure; nerve issues, including carpal tunnel syndrome and nerve damage; history of stroke, brain tumour or brain injury; dizziness, palpitations or fainting; diabetes; current or previous diagnosis of psychiatric condition; use of medication known to alter breathing, blood pressure or mood; pregnancy or breast-feeding; heavy use of tobacco; frequent migraines; epilepsy; and history of concussion resulting in loss of consciousness. Participants taking part in the TMS session (ses-08) were also screened for contraindications to TMS^34^. The study was approved by the Cardiff University School of Psychology Research Ethics Committee (EC.18.08.14.5332RA3). All participants gave informed written consent and gave permission for their anonymised data to be shared with researchers in other organisations and deposited in publicly accessible databases. Participants were told they were free to withdraw from the study at any time.

Details of the equipment and protocols employed as part of WAND are included in the *Equipment* and *Data acquisition* sections below and are organised according to the sessions outlined in Fig. 4. A summary of the data acquired as part of each imaging session is included in Table S2, with summaries of MRI parameters provided in Table S3. In addition, summaries of the cognitive tests and self-reported trait measures used for WAND are included in Table S4, with summary statistics for each trait questionnaire and cognitive test provided in Tables S5 and S6, respectively.

### Equipment

Data were acquired using the following equipment.

#### Magnetoencephalography (ses-01)

Whole-head MEG recordings were acquired at a 1200 Hz sampling rate on a 275-channel CTF radial gradiometer system. An additional 29 reference channels were recorded for noise cancellation purposes and the primary sensors were analysed as synthetic third-order gradiometers^35^. Head digitization was performed prior to the participant entering the magnetically shielded room, using a Polhemus digitising system.

#### Ultra-strong gradient 3 T MRI (ses-02)

Diffusion and quantitative MRI data were acquired using an ultra-strong magnetic field gradient (300 mT/m) 3 T Connectom MRI scanner, modified from a 3 T Magnetom Skyra (Siemens Healthcare, Erlangen, Germany), and a 32-channel receive-only head coil (same vendor). Compared with gradient hardware available on conventional MR systems (45–80mT/m), the Connectom’s ultra-strong magnetic gradients allow for stronger diffusion weighting per unit time, shortening the minimum echo time, improving signal to noise ratio - especially important at higher b-values - and increasing sensitivity to small water displacement^5,36^.

#### 3 T Prisma MRI (ses-03, ses-05, ses-07)

Structural, functional and spectroscopy data for sessions 3, 5 and 7 were acquired using a 3 T Magnetom Prisma system (Siemens Healthcare, Erlangen, Germany), and a 32-channel receive-only head coil.

#### 7 T MRI (ses-04, ses-06)

Ultra-high field structural, functional and spectroscopy data for sessions 4 and 6 were acquired using a Siemens 7 T Magnetom system (Siemens Healthcare, Erlangen, Germany). A 32-channel receive, volume transmit head coil was used (Nova Medical, Wilmington, MA, United States).

#### Physiological monitoring equipment (ses-03, ses-06, ses-07)

Physiological monitoring was conducted in 3 T and 7 T functional sessions using a modified version of the ADInstruments (Oxford, UK) finger pulse transducer, nasal cannula (Salter Labs, London, UK) with respiratory gas analyser (ML206, ADInstruments Ltd, Oxford, UK), and in-house built respiratory belt, recorded using the PowerLab and LabChart data acquisition system (Powerlab 16/35) and LabChart data analysis software (ADInstruments Ltd, Oxford, UK).

#### Transcranial magnetic stimulation (ses-08)

Transcranial magnetic stimulation (TMS) was applied with a Magstim (Whitland, UK) BiStim2 in conjunction with a standard D70 (70 mm, figure of 8) Alpha Flat Coil. These were controlled with Cambridge Electronic Design Limited (CED, Cambridge, UK) Signal software (Version 6.04a), operating via a CED 1401 power acquisition interface, with a Digitimer D440-4 Isolated Amplifier (Digitimer Ltd, Hertfordshire, UK) and pre-gelled Ag/AgCl electrodes for electromyographic recordings. Electrodes were placed between the right index lumbrical and the first dorsal interosseous with a medial epicondyles reference. Coil positioning was maintained with a Manfrotto (Cassola, Italy), tripod and articulated arm. Neuronavigation was performed using a BrainSight (Rogue Research Inc., Montreal, Quebec) and Polaris optical unit (Northern Digital, USA), in conjunction with the participant-specific ses-03 T1-weighted anatomical scan (see below for details).

#### Cognitive testing equipment (non-imaging sessions)

Participant demographic data, cognitive tasks, and the Mini International Neuropsychiatric Interview (MINI) were administered on Day 1 of testing. Participants’ demographics and MINI responses were recorded on the experimenter’s computer using MediaLab software. Computerised cognitive tasks were completed by participants on a separate computer, using PsychoPy (v3.0.4)^37^.

Trait questionnaires (detailed in Table S4 and in the Data acquisition section) were administered on Day 2 of testing. Questionnaires were completed by participants on the same computer, using MediaLab.

### Data acquisition

Acquisition details for each session are provided in this section. For MRI protocols (sessions 2 through 7), we provide only brief details of acquisition parameters here (and in Table S3). Readers are directed to the WAND git repository (https://git.cardiff.ac.uk/cubric/wand.git) for full MRI protocol files.

All MRI data with in-plane undersampling included in this work were reconstructed using GRAPPA^38^-based image reconstruction provided by the vendor, including correction for gradient non-linearities.

#### Magnetoencephalography data (ses-01)

MEG (ses-01) was conducted prior to any MRI, to avoid residual magnetization of tissue following exposure to the MRI scanners’ magnetic fields^39^. Subjects were seated upright in a magnetically shielded room with their head supported by a chin rest to minimise movement. The following additional recordings were acquired: horizontal and vertical electro-oculograms (EOG, to monitor eye blinks and eye movements), bilateral wrist electrocardiogram (ECG), right index finger electromyography (EMG; for the motor task). Ground and reference electrodes were placed on opposite elbows.

MEG tasks were run in the following order: (1) a 10-minute resting state paradigm, (2) a visual gamma task, (3) a Simon cognitive task, (4) an auditory/finger abduction task and (5) a mismatch negativity (MMN) task.

*Resting state*: Participants were asked to sit still and relax, and to focus on a white fixation dot (0.2 degrees in diameter) presented on the screen. The duration of the resting-state recording was 10 min.

*Visual task*: The visual task consisted of 100 trials and was based on a commonly-used strong visual gamma inducing design^40,41^. For each trial, a white fixation dot (0.05 degrees in diameter) was presented for 1.5 s, then a circular sinusoidal grating with spatial frequency 1.5 cycles per degree was presented with the grating contracting towards the centre at two thirds of a degree per second. After a random interval between 0.75 and 3 s, the stimulus speed doubled. The participants were instructed to indicate that they detected a speed change by pressing a button with the right index finger. The grating was removed from the screen 0.5 seconds after the speed change. Feedback was given to the participants about the response timing (“Too soon”, “Too late”, or “OK”) for 1.5 s.

*Simon task*: The Simon task^42^ is a choice reaction time task used to measure response inhibition. Participants were shown a blue or green circle, presented on the left or right of a central fixation cross. Participants were asked to respond by pressing the left button when the blue circle was presented on the screen and the right button when the green circle was presented. Fifty percent of trials were congruent, when the circle appears on the same side as the required response (e.g., a blue circle on the left of fixation), and 50% were incongruent, when the location of the circle is on the opposite side to the correct response (e.g., a blue circle on the right of fixation). Participants completed 8 practice trials followed by 400 experimental trials. Stimuli were presented for 1000 ms with an inter-trial interval that varied between 1500 ms and 2000 ms.

*Auditory task*: The auditory task consisted of 60 trials. For each trial, a 40 Hz click (lasting for 1 ms) was played for 4 s. Participants were instructed to indicate the end of the sound by pushing a lever with a swift, sharp abduction of their right index finger. Trials were separated by an inter-trial interval of random duration between 4 and 4.5 s.

*Mismatch negativity (MMN) task*: The MMN task measures a change-specific component of the auditory event-related potential elicited by any discriminable change in auditory stimulation. Here we used the Optimum 1 paradigm, as previously described by Näätänen *et al*.^43^. Stimuli were presented to both ears at a volume of 75 dB. The standard stimuli were harmonic tones composed of sinusoidal partials of 500, 1000, and 1500 Hz respectively, and were 75 ms in duration (including 5 ms rise and fall times). The intensities of the second and third partials were 3 and 6 dB lower, respectively, than the first partial. Deviant tones differed from the standards either in frequency, duration, intensity, perceived sound-source location, or by a gap in the middle of the tone, but were otherwise identical to the standard tones. Two types of stimuli existed for each of the frequency, intensity, and location deviants. Half of the frequency deviants were 10% higher (partials: 550, 1100, 1650 Hz) and the other half were 10% lower (450, 900, 1350 Hz) than the standard tone. Half of the intensity deviants were −10 dB and the other half + 10 dB compared with the standard. A change in the perceived sound-source location was created by introducing an interaural time difference of 800 μs, in either the right or left channel (half each). The duration deviant was 25 ms in duration. The gap deviant was constructed by cutting out 7 ms (1 ms fall and rise times included) from the middle of the standard stimulus, leaving a silent gap. Each sequence commenced with 15 standard tones after which deviant tones were presented every second tone. All five deviants (10% of trials each) were presented in the same sequence, each interspersed by a standard tone (50% of trials). Within each array of 10 tones (including five standard and five deviant tones) each deviant category was presented once, and two deviants of the same category never followed each other. Stimuli were presented at a stimulus-onset-asynchrony of 500 ms in three 5 min sequences (1845 stimuli in total).

#### Diffusion and quantitative 3 T MRI (300 mT/m magnetic field gradients; ses-02)

**Fig. 5:**
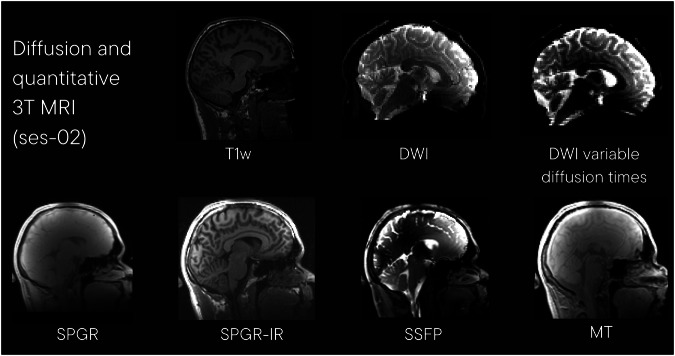
Example images from session 02, Diffusion and quantitative MRI using ultra strong magnetic gradients. T1w: T1-weighted structural image (acquired with MPRAGE); DWI: diffusion weighted image; SPGR: 3D spoiled gradient-recalled echo sequence; SPGR-IR: inversion recovery-prepped spoiled gradient-recalled echo sequence; SSFP: steady-state free precession; MT: quantitative magnetization transfer image.

Diffusion and quantitative myelin sensitive MRI data (ses-02) were acquired on the Connectom 3 T MRI scanner with ultra-strong magnetic field gradients. Example images from this acquisition are shown in Fig. 5. The session included the following acquisitions:

*T1-weighted anatomical*: Data were acquired using a Magnetization Prepared Rapid Gradient Echo (MPRAGE) sequence with 180° preparation radio-frequency pulse with repetition time (TR) 2300 ms, echo time (TE) 2 ms, flip angle 9°, field of view (FOV) 256 × 256 × 192 mm^3^, voxel size 1 × 1 × 1 mm^3^, inversion time (TI) 857 ms, 2-fold in-plane undersampling and phase-encoding direction anterior to posterior (A≫P).

*Multi-shell diffusion-weighted MRI*: Data were acquired using protocols described by Koller *et al*.^36^ for the Microstructural Image Compilation with Repeated Acquisitions (MICRA) dataset. Data were acquired over 18 min using a single-shot spin-echo, echo-planar imaging sequence. Data were acquired in both anterior to posterior (A≫P) and posterior to anterior (P≫A) phase-encoding directions. A≫P data comprised two shells (b = 200 s/mm^2^ and b = 500 s/mm^2^) with 20 diffusion encoding directions (uniformly distributed according to Jones *et al*.^44^), one shell (b = 1200 s/mm^2^) with 30 directions and three shells (b = 2400 s/mm^2^, 4000 s/mm^2^ and 6000 s/mm^2^) with 61 directions, in addition to two leading non-diffusion-weighted (b = 0 s/mm^2^) images and 11 non-diffusion-weighted images dispersed throughout (33^rd^ volume and every 20^th^ volume thereafter). P≫A data comprised two leading non-diffusion-weighted images, one shell of 30 directions at b = 1200 s/mm^2^ and a final non-diffusion-weighted image. Data acquisition details for all b-values were as follows: TR 3000 ms, TE 59 ms, FOV 220 × 220 × 132 mm^3^, voxel size 2 × 2 × 2 mm, with 2-fold in-plane undersampling. Diffusion gradient duration (δ) and separation (∆) were 7 ms and 24 ms, respectively.

*Diffusion-weighted imaging with variable diffusion time*: Data were acquired using a series of diffusion-weighted echo-planar imaging sequences. Data were acquired in both A≫P and P≫A phase-encoding directions. For A≫P data, diffusion encoding gradients were uniformly distributed in space with 2 increments in gradient amplitude per diffusion time (∆), resulting in the following combinations: ∆ = 18 ms with 30 directions each at b = 2200 and 4400 s/mm^2^, ∆ = 30 ms with 30 directions each at b = 4000 and 8000 s/mm^2^, ∆ = 42 ms with 30 directions each at b = 5800 and 11600 s/mm^2^, ∆ = 55 ms with 30 directions each at b = 7750 and 15500 s/mm^2^. Non-diffusion-weighted images were dispersed throughout each acquisition: two leading and four additional volumes (18^th^ volume and every 16^th^ volume thereafter). An additional single non-diffusion-weighted image was acquired in the P≫A phase-encoding direction. Further acquisition details for all diffusion times and b-values were as follows: TR 3900 ms, TE 80 ms, FOV 220 × 220 × 132 mm^3^, and voxel size 2 × 2 × 2 mm^3^, with 2-fold in-plane undersampling. Diffusion gradient duration (δ) was 7 ms.

*Spoiled and unspoiled gradient echo data*: Data were acquired using sequences implementing the McDESPOT (Multicomponent driven equilibrium single pulse observation of T1 and T2) protocol^45^, including a T1-weighted 3D spoiled gradient-recalled echo sequence (SPGR), an inversion recovery-prepped spoiled gradient-recalled echo sequence (SPGR-IR) and a steady-state free precession (SSFP) sequence. Data acquisition details have been reported previously^36^ and were as follows: for SPGR – TR 4 ms, TE 1.9 ms, 8 flip angles (3, 4, 5, 6, 7, 9, 13 and 18°); for SPGR-IR – TR 4 ms, TE 1.9 ms, flip angle 5°, full k-space acquisition in phase encoding and slice directions; for SSFP – TR 4.54 ms, TE 2.27 ms, 8 flip angles (10, 13.33, 16.67, 20, 23.33, 30, 43.33 and 60°). For all multi-component relaxometry data, phase-encoding direction was A≫P, FOV 220 × 220 × 179 mm^3^, matrix size 128 × 128 × 104 and voxel size 1.72 × 1.72 × 1.72 mm^3^.

*Optimised quantitative magnetization transfer (qMT)*: Data were acquired using a prototype turbo-flash sequence previously described by Koller *et al*.^36^. QMT data included 11 magnetization transfer (MT)-weighted images with flip angle (°)/frequency offset (Hz) combinations of: 628/47180; 332/56360; 628/12060; 332/1000; 333/1000; 628/2750; 628/2770; 628/2790; 628/2890; 2*628/1000 and 1 non-MT-weighted image^46,47^. All data were acquired with TR 55 ms, TE 2.1 ms, FOV 220 × 220 × 179 mm^3^, matrix size 128 × 128 × 104, voxel size 1.72 × 1.72 × 1.72 mm^3^, turbo factor 4, 3-fold in-plane undersampling, phase-encoding direction A≫P, and radial reordering with non-selective excitation magnetization transfer pulse duration 15.36 ms.

#### 3 T functional and perfusion MRI (ses-03)

**Fig. 6:**
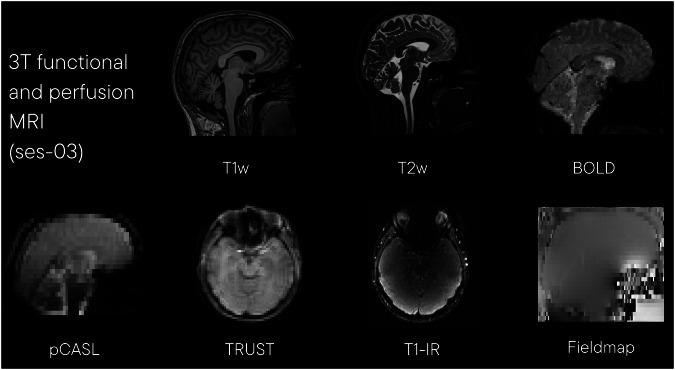
Example images from session 03, 3 T functional and perfusion MRI. T1w: T1-weighted structural image (acquired with MPRAGE); T2w: T2-weighted turbo spin echo; BOLD: blood oxygen level dependent functional MRI; pCASL: pseudo-continuous arterial spin labelling; TRUST: T2 relaxation under spin tagging; T1-IR: T1 inversion recovery.

Functional task and perfusion data (ses-03) were acquired on a 3 T Prisma MRI scanner. Example images from this acquisition are shown in Fig. 6. The session included the following acquisitions:

*T1-weighted anatomical*: Data were acquired using an MPRAGE sequence with TR 2250 ms, TE 3.06 ms, flip angle 9°, FOV 256 × 288 × 176 mm^3^, voxel size 1 × 1 × 1 mm, TI 850 ms, 2-fold in-plane undersampling and phase-encoding direction A≫P.

*T2-weighted anatomical*: Data were acquired using a whole-brain 2D T2-weighted turbo spin echo sequence with TR 8000 ms, TE 82 ms, flip angle 120°, echo train length (turbo factor) 17, echo spacing 8.22 ms, 2-fold in-plane undersampling, 2 concatenations, 88 contiguous 2 mm slices with an in-plane resolution of 1 × 1 mm^2^ and FOV 256 × 256 mm^2^.

*Task-based and resting-state blood oxygen level dependent (BOLD) functional MRI*: Data were acquired using a multiband gradient echo echo planar imaging (EPI) sequence^48–50^ with TR 2000 ms, TE 30 ms, flip angle 70°, echo spacing 0.55 ms, FOV 192 × 192 × 160 mm^3^, voxel size 2.0 × 2.0 × 2.0 mm^3^, 4 simultaneously excited slices, 2-fold in-plane undersampling, and phase-encoding direction A≫P. During these scans, physiological monitoring was conducted using a nasal cannula, respiratory belt, and a pulse ox for measuring heart rate. Functional tasks were as follows:

*Visual category functional localiser (fLoc) task*: Two runs of BOLD fMRI were acquired while participants completed a visual category functional localiser (fLoc) task (modified from Stigliani *et al*.^51^). In this task, 400 grayscale images depicting different stimulus categories (category names shown in parentheses) were presented to the participant, including scenes (‘corridor’), characters (‘word’), bodies (‘body’), faces (‘adult’), and objects (‘car’). Stimuli were presented in 4 s mini-blocks. Within a block, eight images from a given category were presented sequentially (image duration 0.5 s). Each run included 40 presentations of each of the five categories as well as a blank baseline condition (also of 4 s duration). Each run lasted 4 min 48 s. During the task, participants completed a 1-back task, where they were instructed to press a key whenever a stimulus was repeated across two trials. The frequency of repetitions was matched across categories, with no more than 1 repetition per 4 s mini-block.

*Reversal learning task*: One run of BOLD fMRI was acquired while participants completed a reversal learning paradigm, previously described by Lancaster *et al*.^52^. Briefly, participants learned to select one of two simultaneously presented colours (“blue” or “green”), receiving monetary reward for correct choices and monetary punishment for incorrect choices. After 7–11 trials, reward/punishment contingencies were reversed so that the previously rewarded colour was now punished, and vice versa. Within each reversal episode, one or two probabilistic error trials were included, in which “wrong” feedback was given for correct choices. The paradigm lasted 12 min 26 s.

*Resting state*: One run of BOLD fMRI was acquired while participants were at rest. Resting state fMRI was acquired over 10 min 38 s; participants were instructed to look at a white fixation cross presented at the centre of their field of view, with a black background.

*3D phase contrast*: Data were acquired for planning the following pseudo-continuous arterial spin labelling (pCASL) acquisitions, with v_enc_ 75 cm s^−1^; TR 40.65 ms, TE 5.6 ms, flip angle 10°; FOV 180 × 240 × 78 mm^3^ and voxel size: 0.47 × 0.47 × 1.30 mm^3^.

*M0 scans*: Two M0 images with different phase encoding directions (A≫P and P≫A) for cerebral blood flow quantification, with TR 6000 ms, TE 11 ms, flip angle 90°, 2-fold in-plane undersampling, 22 slices with slice thickness of 5 mm, voxel resolution 3.4 × 3.4 × 5.0 mm, and FOV 320 × 320 mm^2^.

*Pseudo-continuous arterial spin labelling*: Data were acquired for voxel wise quantification of cerebral blood flow^53^, with TR 4600 ms, TE 11 ms, post label delay 2000 ms, tag duration 1800 ms, flip angle 90°, 2 -fold in-plane undersampling, 22 slices of 2D EPI with a slice thickness of 5 mm, voxel resolution 3.4 × 3.4 × 5.0 mm and FOV 320 × 320 mm^2^.

*T2 relaxation under spin tagging (TRUST)*: Data were acquired to estimate venous blood oxygenation in the superior sagittal sinus^54^. Venous blood oxygenation can be combined with the pCASL measurement of cerebral blood flow using the Fick principle^55^ for a global estimate of cerebral metabolic rate of oxygen consumption. A single slice, angled parallel to the anterior commissure - posterior commissure (AC-PC) line, was acquired, cutting through the superior sagittal sinus 20 mm superior to the confluence of sinuses. Acquisition parameters matched those previously reported by Jiang *et al*.^56^, including TR 3000 ms, TE 3.9 ms, four effective echo times of TE 0, 40, 80, and 160 ms, with a t_CPMG_ 10 ms, echo spacing 0.49 ms, 3-fold in-plane undersampling, FOV 220 × 220 mm^2^, voxel size 3.4 × 3.4 × 5 mm, TI 1020 ms, phase partial Fourier 6/8. Three repetitions were acquired.

*T1 inversion recovery*: Data were acquired to calculate haemoglobin in the superior sagittal sinus, with TR and ΔTR 150 ms, TE 22 ms, post label delay 1800 ms, flip angle 90°, 2-fold in-plane undersampling, FOV 240 × 240 mm^2^, and 1 slice with slice thickness 3 mm. The slice position centre and orientation matched the TRUST scan.

*Gradient echo field maps* were acquired using a multi echo gradient echo sequence with TR 434 ms, TE_1_ 4.92 ms, TE_2_ 7.38 ms, flip angle 60°, voxel resolution 2.0 × 2.0 × 6.0 mm^3^, and FOV 96 × 96 mm^2^.

#### 7 T magnetic resonance spectroscopy (ses-04)

For optimal separation of glutamate, glutamine and myo-inositol peaks, *in vivo* MRS data (ses-04) were acquired on a Siemens Magnetom 7 T investigational device using a semi-LASER (semi-localization by adiabatic selective refocusing) acquisition sequence^57–59^. The session included the following acquisitions:

*B1 inhomogeneity map*: Data were acquired using a 3-dimensional dual refocusing echo acquisition mode (3DREAM) sequence^60^ with TR 5000 ms, TE_1_ 0.9 ms, TE_2_ 1.55 ms, flip angle_1_ 60°, flip angle_2_ 8°, flip angle_3_ 3°, FOV 200 × 200 × 200 mm^3^, matrix size 64 × 64 × 64, voxel size 4.5 × 4.5 × 4.5 mm^3^, and phase-encoding direction R≫L.

*T1-weighted anatomical*: Data were acquired using an MPRAGE sequence with TR 2200 ms, TE 3.02 ms, flip angle 7°, FOV 224 × 224 × 157 mm^3^, voxel size 0.7 × 0.7 × 0.7 mm^3^, TI 1050 ms, 2-fold in-plane undersampling, phase-encoding direction A≫P, and a TR-FOCI adiabatic inversion pulse^61^.

*Metabolite and water reference data*: MRS data were acquired from 4 volumes of interest (VOIs), including the left sensorimotor cortex (30 × 30 × 30 mm^3^; voxel positioned to cover the hand knob of the left sensorimotor area), anterior cingulate cortex (25 × 30 × 40 mm^3^; voxel angled to run parallel to the corpus callosum and avoiding the lateral horns of the ventricles), occipital cortex (30 × 30 × 30 mm^3^; voxel positioned parallel to the straight sinus and ensuring the scalp is excluded) and right auditory cortex (30 × 45 × 20 mm^3^; voxel positioned parallel with and inferior to the Sylvian fissure). Before acquisition of each VOI, linear and higher order shims were used to optimise the static magnetic field (B_0_) within the VOI, using 2 to 3 iterations of automatic shimming (using the vendor-provided optimisation using gradient echo field maps) – this process was repeated if the full-width-half-maximum (FWHM) of the magnitude spectrum of the water peak was > 30 Hz. Data acquisition for all VOIs were as follows: TR 5230 ms, TE 78 ms, 64 excitations per spectrum, spectral width 3000 Hz, number of spectral points 2048, frequency offset −1.7 ppm for the metabolite spectrum and 0 ppm for the water reference spectrum, water suppression using Variable Power and Optimized Relaxations delays (VAPOR^62^).

#### 3 T edited magnetic resonance spectroscopy (ses-05)

For optimal separation of the γ-amino-butyric acid (GABA) from overlapping resonances, GABA-edited nuclear MRS data (ses-05) were acquired on a Siemens Magnetom Prisma 3 T scanner using a Meshcher-Garwood Point RESolved Spectroscopy (MEGA-PRESS)^63,64^ sequence. The session included the following acquisitions:

*T1-weighted anatomical*: Data were acquired using an MPRAGE sequence with TR 2250 ms, TE 3.06 ms, FOV 256 × 288 × 176 mm^3^, voxel size 1 × 1 × 1 mm^3^, flip angle 9°, TI 850 ms, 2-fold in-plane undersampling and phase-encoding direction A≫P.

*GABA-edited MRS and water reference data*: Data were acquired from 4 VOIs, including the left sensorimotor cortex (30 × 30 × 30 mm^3^), anterior cingulate cortex (40 × 30 × 25 mm^3^), occipital cortex (30 × 30 × 30 mm^3^) and right auditory cortex (45 × 30 × 20 mm^3^). Voxel position was identical to the 7 T protocol (ses-04). Before acquisition of each VOI, linear and higher order shims were used to optimise the static magnetic field (B_0_) within the VOI, using 2 to 3 iterations of automatic shimming (Siemens’s *Brain* shimming mode). This process was repeated if the FWHM of the magnitude spectrum of the water peak was > 20 Hz. Data acquisition for all VOIs were as follows: TR 2000 ms, TE 68 ms, 160 excitations per spectrum, spectral width 2000 Hz, number of spectral points 2048, frequency offset −1.7 ppm for the metabolite spectrum and 0 ppm for the water reference spectrum water suppression using VAPOR^62^. Editing pulses were positioned at 1.9 and 7.5 ppm at a bandwidth of 100 Hz.

#### 7 T structural and function MRI (ses-06)

**Fig. 7:**
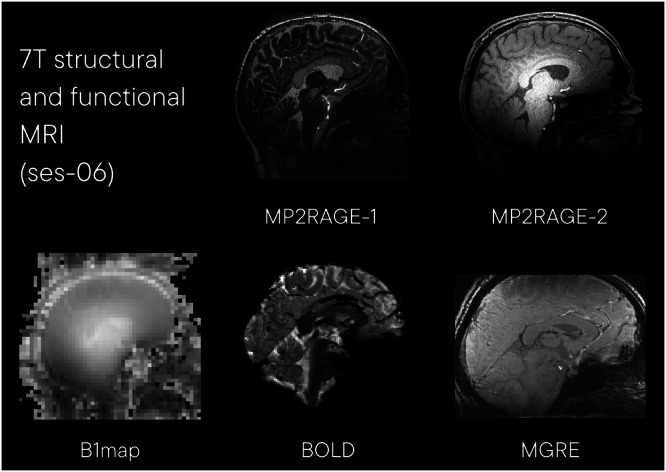
Example images from session 06, 3 T functional and perfusion MRI. MP2RAGE: magnetization prepared 2 rapid gradient echoes; B1map: B1 inhomogeneity map; BOLD: blood oxygen level dependent functional MRI; MGRE: Multi-echo 3D gradient echo.

High resolution structural and function MRI data (ses-06) were acquired on a Siemens Magnetom 7 T investigational device. Example images from this acquisition are shown in Fig. 7. The session included the following acquisitions:

*B1 inhomogeneity map*: Data were acquired using a 3DREAM sequence^60^ with TR 5000 ms, TE_1_ 0.9 ms, TE_2_ 1.55 ms, flip angle_1_ 60°, flip angle_2_ 8°, flip angle_3_ 3°, FOV 200 × 200 × 200 mm^3^, matrix size 64 × 64 × 64, voxel size 4.5 × 4.5 × 4.5 mm^3^, and phase-encoding direction R≫L.

*T1-weighted anatomical*: Data were acquired using a magnetization prepared 2 rapid gradient echoes (MP2RAGE) sequence with TR 3500 ms, TE 2.64 ms, FOV 224 × 224 × 157 mm^3^, voxel size 0.7 × 0.7 × 0.7 mm^3^, flip angle_1_ 5°, flip angle_2_ 2°, TI_1_ 725 ms, TI_2_ 2150 ms, 3-fold in-plane undersampling, phase-encoding direction A≫P and a TR-FOCI adiabatic inversion pulse^61^. For radiofrequency transmit power calibration, we employed the protocol proposed by the UK 7 T Network^65^. Transmit voltage was calculated based on a 3DREAM B1 + map (see above), with a voltage scaling factor based on mean flip angle over a single axial slice, where the brain is longest in the A≫P direction, scaling to 80% of the targeted 60° flip angle.

The MP2RAGE parameters were optimized for phase-sensitive inversion recovery contrast^66^, such that grey and white matter have opposite phase at the first inversion. Image reconstruction for the MP2RAGE was performed using code developed by the UK 7 T Network^65^, using a uniform sensitivity Roemer array coil combination^67,68^ with coil sensitivities calculated using two 3D gradient echo acquisitions acquired immediately prior to the MP2RAGE (one using the 32-channel array, the other using the volume coil) with TR 6 ms, TE 1.52 ms, flip angle 5°, FOV 360 × 360 × 360 mm^3^, voxel size 5.6 × 5.6 × 5 mm^3^, and bandwidth 1000 Hz/px.

*T2-weighted anatomical*: Data were acquired using a 2D T2-weighted turbo spin echo sequence, oriented orthogonal to the long axis of the hippocampus with TR 13000 ms, TE 75 ms, flip angle 120°, echo train length (Turbo factor) 9, in-plane resolution 0.4 × 0.4 mm^2^, FOV 220 × 220 mm^2^, 2-fold in-plane undersampling, and echo spacing 15.1 ms. In-plane interpolation was performed during reconstruction to 0.2 × 0.2 mm^2^ in-plane resolution. Distortion correction was performed to correct for gradient non-linearities using the vendor-provided online reconstruction option. These image parameters were selected to meet the requirements of gold-standard hippocampal subfield and medial temporal lobe automated segmentation tools (e.g., Automatic Segmentation of Hippocampal Subfields, ASHS^69^) and associated subfield atlases^70^.

*Resting-state BOLD functional MRI*: Data were acquired over 10 minutes (400 volumes) using a multiband gradient echo EPI sequence^48–50^ with TR 1500 ms, TE 25 ms, flip angle 65°, echo spacing 0.72 ms, voxel size 1.5 × 1.5 × 1.5 mm^3^, FOV 192 × 192 mm^2^, multiband acceleration factor 4, 2-fold in-plane undersampling, phase partial Fourier 6/8, and phase encode direction A≫P. During the scan, participants were instructed to look at a white fixation cross, presented at the centre of their field of view, with a grey background. Physiological monitoring was conducted with a pulse oximeter, nasal canula, and respiratory belt and was recorded with LabChart software (ADInstruments Ltd, Oxford, UK).

To enable distortion correction of the EPI data^71^, two spin echo EPI datasets were acquired with matched EPI readout timings and geometry to the resting state dataset, one with matched phase encode direction (A≫P) and the other with reversed phase encode direction (P≫A). Three volumes were acquired for each, with TR 2000 ms, TE 45 ms. These were acquired immediately preceding the resting state fMRI.

*Multi-echo 3D gradient echo*: Data were acquired with multi-channel phase combination performed using the ASPIRE method^72^ with TR 39 ms, flip angle 11°, 2 × 2 undersampling, voxel size 0.67 × 0.67 × 0.67 mm^3^, FOV 224 × 224 × 224 mm^3^, 192 slices, and bandwidth 290 Hz/px. Seven echoes were acquired with a monopolar readout and echo times between 5 and 35 ms in 5 ms increments.

#### 3 T metabolic MRI (ses-07)

**Fig. 8:**
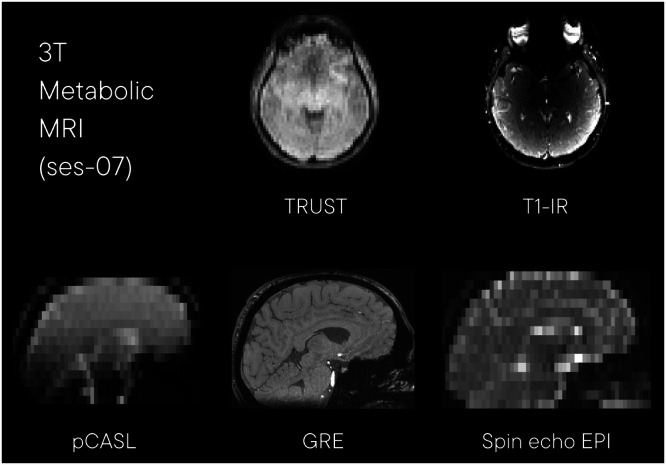
Example images from session 07, 3 T functional and perfusion MRI. TRUST: T2 relaxation under spin tagging; T1-IR: T1 inversion recovery; pCASL: pseudo-continuous arterial spin labelling; GRE: Single-echo 3D gradient echo sequence; EPI: echo planar image.

A subset of 39 participants returned two weeks after their initial visit for an additional MRI scan to measure various aspects of cerebral metabolism (ses-07). Example images from this acquisition are shown in Fig. 8. The session took place on day 4 of testing and included the following acquisitions:

*T2 relaxation under spin tagging (TRUST)*: Data were acquired to estimate venous blood oxygenation in the superior sagittal sinus^54^, a repetition of the measurement acquired in *ses-03*. This measurement was repeated in this session for direct comparison with other measurements in the same session, accounting for natural physiological variability in cerebral perfusion and metabolism that could occur between the timepoints of *ses-03* and *ses-07*. A single slice, angled parallel to the anterior commissure - posterior commissure (AC-PC) line, was acquired, cutting through the superior sagittal sinus 20 mm superior to the confluence of sinuses. Acquisition parameters matched those previously reported by Jiang *et al*.^56^, including TR 3000 ms, TE 3.9 ms, four effective echo times of TE 0, 40, 80, and 160 ms, with a t_CPMG_ 10 ms, echo spacing 0.49 ms, 3-fold in plane undersampling, FOV 220 × 220 mm^2^, voxel size 3.4 × 3.4 × 5 mm^3^, TI 1020 ms, and phase partial Fourier 6/8. Three repetitions were acquired.

*T1 inversion recovery*: Data were acquired to calculate haemoglobin in the superior sagittal sinus, a repetition of the measurement acquired in *ses-03*. Parameters were as follows: TR and ΔTR 150 ms, TE 22 ms, post label delay 1800 ms, flip angle: 90°, 2-fold in-plane undersampling, FOV 240 × 240 mm^2^, and 1 slice with slice thickness 3 mm. The slice position centre and orientation matched the TRUST scan.

*3D phase contrast*: Data were acquired for planning the following pseudo-continuous arterial spin labelling (pCASL) acquisitions, with v_enc_ 75 cm s^−1^, TR 40.65 ms, TE 5.6 ms, flip angle 10°, FOV 180 × 240 × 78 mm^3^ and voxel size: 0.47 × 0.47 × 1.30 mm^3^.

*Simultaneous BOLD and ASL cerebral blood flow data*: Data were acquired using an in-house dual-excitation^73^ (*DEXI*) pCASL sequence with two inversion pulses for background suppression. The labelling duration and the Post Label Delay (PLD) were both set to 1.5 s, 3-fold in-plane undersampling was used with TE_1_ = 10 ms and TE_2_ = 30 ms. A TR of 4.4 s was used to acquire 15 slices of 2D EPI, in-plane resolution 3.4 × 3.4 mm and slice thickness 6 mm (33% slice gap). This 10 min DEXI protocol was repeated three times: (i) during a breath hold task, with grey background; (ii) resting state, with the participant fixating on a cross at the centre of the field of view; (iii) during a breath hold task, with an isoluminant 8 Hz reversing radial checkerboard in the background, sustained throughout. The breath hold task consisted of 10 repeats of a 20 s breath hold, with visual prompts at the centre of the participant’s field of view and the breath hold starting at the end of exhalation. In between breath holds in the breath hold task, participants were visually cued to pace their breathing at 3 s inhale, 3 s exhale. For four participants, this pacing was set as 1.6 s inhale, 2.4 s exhale, however the higher breathing frequency resulted in hyperventilation, so the participants were mildly hypocapnic. Therefore, the remaining 35 participants were cued at 3 s inhale, 3 s exhale and did not hyperventilate.

*M0 scans*: For each DEXI dataset, a pair of M0 scans was acquired with opposite phase encoding directions (A≫P and P≫A) for cerebral blood flow quantification with TR 6000 ms, TE 10 ms, flip angle 90°, 3-fold in-plane undersampling. EPI parameters and resolution matched the DEXI dataset.

*Spin-echo EPI*: In addition to dual-excitation pCASL for measuring cerebral blood flow, a spin-echo EPI sequence with breath-hold task was acquired, with TR 2500 ms, TE 90 ms, echo spacing 0.55 ms, voxel size 3.4 × 3.4 × 6.5 mm^3^, 15 slices, with 3-fold in-plane undersampling. The breath hold task was identical to that performed during the DEXI acquisition.

*Single-echo 3D gradient echo sequence*: The session also included a single-echo 3D gradient echo sequence for quantitative susceptibility mapping and vein localisation. Acquisition parameters consisted of TR 20 ms, TE 14 ms, flip angle 15°, voxel size 0.8 × 0.8 × 1.0 mm^3^, FOV 230 × 230 × 144 mm^3^, 144 slices, 2-fold in-plane undersampling, and bandwidth 220 Hz/Px^53,74^.

#### Transcranial magnetic stimulation (ses-08)

A subset of 40 participants underwent TMS (ses-08), where the primary experiment quantified Motor Threshold (MT), Short and Long Interval Intra Cortical Inhibition (S/LICI)^75,76^ The TMS session took place on day 3 of testing.

During the TMS session, the participant’s head position was registered to a T1 image using the BrainSight system. Electromyographic (EMG) equipment was applied and checked with a hand clench. Throughout the experiment the coil was positioned over the left primary motor region with the handle pointing posteriorly and rotated at 45 degrees to the midline^77^. The ‘hot-spot’ was identified with single-pulse stimulation with an initial intensity set to 45% of the maximum and the coil placed over the visually identified hand knob of the left precentral gyrus. The intensity was then increased (or decreased) by 5% until a motor twitch was observed. The coil was then repeatedly moved forward, left, backwards and right and rotated clockwise and anticlockwise until the twitch appeared optimal, which was then marked using the BrainSight as the ‘hot-spot’. This was targeted throughout the rest of the experiment within a 5 mm tolerance. A staircase procedure was then applied to determine MT with a motor response criteria of a reliable 0.2 mV EMG deflection^78–80^ suitable for combination with S/LICI assay. During this, TMS was initially set to approximately 10% below that used to determine the hot-spot. Then, if 10/10 responses were not registered, the stimulator intensity was increased in 5% steps until 10/10 were registered. Then, intensity was decreased by 2% until there were less than 10 responses, and finally, intensity was increased by 1% until 10/10 responses were observed.

S/LICI involved paired pulses consisting of a conditioning pulse at 70% of MT^81,82^ followed by the test MT pulse at interstimulus intervals of 1, 3, 5, 7 and 15 ms. Both the conditioning pulse and MT pulse were also administered in isolation (7 conditions in total). We aimed to collect four blocks of 70 trials (280 trials in total, 40 trials per condition) where the trial order was pseudorandomised for each block. Thirty-five participants completed full blocks, and 5 completed three blocks.

#### Participant demographics, neuropsychiatric interview, and cognitive data

Participant demographics, the MINI neuropsychiatric interview, and cognitive testing took place on Day 1, in a cognitive lab. Where specified, cognitive tests included tasks from the Wechsler Memory Scale IV (WMS-IV^83^) and the Wechsler Abbreviated Scale of Intelligence II (WASI-II^84^). Participants gave consent for a tape recorder to be used where necessary.

*Participant demographics*: Data included in the WAND data release^22^ include date of birth, participant sex, height, weight, waist and hip circumference, blood pressure and self-reported consumption of alcohol and cigarettes.

*Mini International Neuropsychiatric Interview (MINI)*: The MINI^85^ was administered to participants to ensure the absence of personal history of psychosis or related disorders, current or recent depressive episodes, or other conditions that may significantly affect the results of the study. The sections on suicidality, anorexia nervosa and bulimia nervosa were omitted.

*Choice reaction-time task 1*: Participants were instructed to quickly and accurately categorise black and white images as either a face (z key) or a scene (m key). Stimuli were taken from the fLoc functional localiser package^51^, but not shown in the functional localizer fMRI task (ses-03). Each stimulus was presented until a response was given or for a maximum of 1500 ms, with a 750 ms inter-trial interval. Participants initially completed 10 practice trials, for which they received feedback about their accuracy. Participants then completed 408 experimental trials without feedback, divided into three blocks.

*Logical memory task part 1 (WMS-IV)*: Participants were read two short stories by the experimenter and were asked to recall details of them immediately after each one. The responses of the participants were recorded on a tape recorder.

*Vocabulary (WASI-II)*: Participants were asked to describe the meaning of up to 28 words that were presented both visually and spoken by the experimenter. The words got progressively more difficult. The participant responses were recorded on a tape recorder for offline verification. The test was discontinued in the event of three consecutive incorrect or missed responses.

*Matrix reasoning (WASI-II)*: Participants completed the matrix reasoning subtest of the WAIS-II. Participants were shown up to 27 stimuli that consisted of an incomplete visual matrix or sequence and were asked to select the item that completes the matrix/sequence from a set of 5 items. The test was discontinued in the event of three consecutive incorrect or missed responses. An estimate of full-scale IQ can be obtained from the two WASI-II subtests.

*Verbal fluency*: Participants were asked to name as many things as they could in 60 seconds, in the following categories: things beginning with the letter ‘f’, animals, names of their friends, things in their bedroom^86^. Time was kept with a stopwatch and the experimenter recorded the answers on a tape recorder.

*Logical memory task part 2 (WMS-IV)*: Participants were asked to recall details of the two stories they were read in part 1 of this task, without the stories being read to them again. Participants were also given 30 (15 per story) yes or no questions about the stories. The responses were recorded on a tape recorder.

*Choice reaction time task 2*: This was a four-choice reaction time task based on previous studies^87,88^. Four empty circles were horizontally distributed on the screen. On each trial, one of the circles was filled white and participants were asked to quickly and accurately press a key (c, v, b or n) corresponding to the location of the filled circle. Each stimulus was presented until a response was given, with a 750 ms inter-trial interval. Participants completed eight practice trials with feedback, followed by 372 experimental trials divided into three blocks.

*Balloon analogue risk task (BART)*: The BART is designed to measure risk-taking behaviour^89^. Participants were presented with a sequence of 30 balloons on screen. They were given the opportunity to ‘pump up’ the balloon to earn points (5 points per ‘pump’). They could bank the points at any point by pressing the ‘Enter’ key. Each balloon had a ‘bursting’ point that was unknown to the participant (mean = 65 pumps), which if reached meant the participant received no points for that trial. Thus, participants had to balance risk with reward.

#### Trait questionnaire data

Trait questionnaire data were acquired on Day 2 of testing and included the following:

*Short UPPS-P*: The short version of the Urgency-Premeditation-Perseverance-Sensation Seeking-Positive Urgency (UPPS-P)^90^ consists of 20 items that measure five sub-facets of impulsive behaviour. *Positive urgency* and *negative urgency* reflect a tendency to act rashly when experiencing positive and negative emotions, respectively. *Lack of premeditation* reflects a tendency to act without forethought. *Lack of perseverance* reflects a tendency to lose focus on a task. *Sensation seeking* reflects a tendency to seek novel and exciting experiences. Higher values on the subscales reflect higher levels of impulsivity. Items are measured on a 4-point scale ranging from “Agree strongly” (1) to “Disagree strongly” (4).

*Generalized Anxiety Disorder 7-item (GAD-7) scale*: The GAD-7^91^ is a screening tool for generalized anxiety disorder. The items ask about the frequency of being bothered by anxiety related-problems and were scored on a 4-point scale ranging from “Not at all” (1) to “Nearly every day” (4). In the original scale, items were scored on a 0 to 3 scale and we rescaled the total score to be consistent with this. Higher scores reflect higher levels of anxiety-related symptoms. An additional 8^th^ item asked participants about the extent to which problems they experienced interfered with their daily lives.

*The Anxiety Depression Distress Inventory-27 (ADDI-27)*: The ADDI-27^92^ consists of 27 items that measure three sub-scales of symptoms relating to anxiety and depression. *General distress* refers to symptoms related to negative affect. *Positive affect* captures experiences of positive emotions, with low scores reflecting anhedonia. Somatic anxiety refers to physical symptoms of anxiety (e.g. trembling). Items asked about the extent to which each symptom was experienced on a 5-point scale ranging from “Not at all” (1) to “Extremely” (5).

*Global physical activity questionnaire (GPAQ)*: The GPAQ^93^ was developed by the World Health Organization as a tool for measuring physical activity in adults. It consists of 16 questions that ask about physical activity in work, travel, and recreational activities. A measure of overall physical activity (in minutes per week) was produced, accounting for the intensity of the activity.

*Emotion regulation questionnaire (ERQ)*: The ERQ^94^ consists of 10 items that measure two subscales corresponding to the use strategies to regulate one’s emotions. *Cognitive reappraisal* refers to the reframing of an emotional situation to change its impact. *Expressive suppression* refers to the inhibition of emotional behaviours. Higher scores reflected greater use of that strategy. Items were scored on a 7-point scale ranging from “Strongly disagree” to “Strongly agree”.

*The big five inventory–2 short form (BFI-2-S)*: The BFI-2-S^95^ consists of 30 items that measure the “big five” personality dimensions: Extraversion, Agreeableness, Conscientiousness, Open-mindedness (sometimes called openness to experience) and negative emotionality (sometimes called neuroticism or emotional (in)stability). Items were scored on a 5-point scale ranging from “Disagree strongly” (1) to “Agree strongly” (5).

*Schizotypal personality questionnaire (SPQ)*: The SPQ^96^ consists of 74 items that measure schizotypal features. It produces a total score and nine subscale scores: *Ideas of reference* (e.g. the belief that people are talking about you), *social anxiety, odd beliefs/magical thinking, unusual perceptual experiences, eccentric/odd behaviour and appearance, no close friends, odd speech, constricted affect, and suspiciousness/paranoid ideation*. Items are dichotomous (yes/no).

*The single-item need to belong scale (SIN-B)*: The SIN-B^97^ is a single-item scale that evaluates the extent to which an individual feels a “need to belong” in the context of interpersonal attachments. The item is scored on a 5-point scale ranging from “Strongly disagree” to “Strongly agree”.

*The Survey of Autobiographical Memory (SAM)*: The SAM^98^ is a 26-item self-report measure of memory functioning across four domains: *episodic memory, semantic memory, spatial memory*, and *future prospection*. Items are scored on a 5-point scale ranging from “Strongly disagree” to “Strongly agree”. The recommended scoring of the SAM involves an unpublished set of weights available on request from the authors of the scale. We instead computed average scores for each subscale, which have been shown to correlate well with scores produced by the recommended procedure^99^.

*The navigational strategies questionnaire (NSQ)*: The NSQ^100^ measures the extent to which individuals adopt a map-based navigation strategy as compared to a non-map-, or scene-, based strategy. The scale consists of 14 dichotomous or trichotomous items. The score reflects a tendency to use a map-based strategy calculated as the difference between the number of map-based and non-map-based answers given.

*The mind wandering questionnaire (MWQ)*: The MWQ^101^ is a 5-item scale that measures the extent to which individuals experience disruption from task-unrelated thoughts. Items are scored on a 6-point scale ranging from “Almost never” (1) to “Almost always” (6).

*The fazio laterality inventory (FLI)*: The FLI^102^ is a 10-item scale that measures handedness. Participants are asked to indicate the percentage of time that they use their right hand for various tasks (e.g. writing). Higher scores reflect a greater right-hand preference. Two additional items ask whether participants self-identify as right-handed, left-handed or ambidextrous; and whether answers are influenced by any injuries or impairments.

*The hospital anxiety and depression scale (HADs)*: The HADs^103^ is a 14-item scale that measures two subscales corresponding to symptoms of *anxiety* and *depression*. Items probe the frequency or severity of the symptoms on a 4-point scale (with varying text anchors).

*The visual vertigo analogue scale (VVAS)*: The VVAS^104^ is a 9-item scale assessing the extent to which individuals experience symptoms of dizziness in different scenarios (e.g. in a supermarket or travelling in a car). The amount of dizziness in each scenario is rated on an 11-point scale ranging from “no dizziness” (0) to “the most dizziness” (10).

*The adult autism spectrum quotient, short version (AQ-Short)*: The AQ-Short^105^ is a 28-item self-report measure of autistic traits. The scale produces a total score and five subscale scores: *difficulties with social skills, a preference for routine, attention-switching difficulties, difficulties in imagination*, and *a fascination with numbers/patterns*. Items are scored on a 4-point scale ranging from “Definitely agree” (1) to “Definitely disagree” (4). Higher scores correspond to higher levels of autistic traits.

*Visual discomfort images*: Participants rated the extent to which they experienced visual discomfort when viewing 20 static images of abstract art, geometric shapes and buildings^106,107^. Images were classified as either *high discomfort* or *low discomfort*. Visual discomfort was rated on an 11-point scale ranging from “no discomfort” (0) to “high discomfort” (10).

*Adolescent/Adult Sensory Profile (AASP)*: The AASP^108^ is a 60-item scale that measures sensory processing. It produces four subscales corresponding to Dunn’s (1997) model^109^ of sensory processing: *low registration, sensation seeking, sensory sensitivity* and *sensation avoiding*. Participants rate the frequency of behaviours in relation to sensory experiences (e.g. touch, taste) on a five-point scale ranging from “Almost never” to “Almost always”.

*Migraine screening questionnaire (MS-Q)*: The MS-Q^110^ is a 5-item questionnaire developed as a screening tool for migraines. Participants indicate whether they experience each symptom on a dichotomous scale (No/Yes).

### Pre/post-processing of data

To protect the identity of participants, all MPRAGE data have been defaced using the FMRIB Software Library (FSL)^111^ deface tool. Defacing of MRI data has been shown to have potentially deleterious effects on brain segmentation^112^ and assessments of tissue volume^113,114^. To mitigate these effects, we also provide high quality brain-extracted T1w images, extracted using HD-BET^115^. These can be found in the derivatives directory.

Quality reports were generated for multi-shell high angular resolution diffusion imaging (ses-02) using FSL’s eddy-qc tool^116^, as well as T1w (ses-02, ses-03, and ses-04) and T2w structural data (ses-03, and ses-06), and blood-oxygen level dependent functional tasks (ses-03, ses-06 and ses-07) using MRIqc^117^ and MRIQCEPTION (https://github.com/elizabethbeard/mriqception). Quality reports can be found in the derivatives directory or can be generated using scripts provided in the code folder of the git repository.

To facilitate use of the MP2RAGE data (ses-06), we also provide processing code for combining the magnitude and phase data for both inversion times into (i) the phase-sensitive inversion recovery (PSIR) contrast, which can be input into segmentation and image co-registration pipelines and (ii) a T1 mapping calculation based on a Bloch-simulation look-up table. These initial processing outputs are also being released as derivatives. (i) PSIR was calculated as the polarity-restored ratio of the first inversion signal magnitude (|S_1_|) and the sum of magnitudes of both first and second inversion signals (|S_1_| + |S_2_|), see equation 1 of Mougin 2016^66^. This PSIR data ranges between ±1, with white matter signal being positive and grey matter signal being negative. For application in standard image segmentation and registration, a background noise correction was also performed^118^, giving a noise-corrected PSIR image, which appears similar to a standard T1-weighted MRI. (ii) Further, a B1-corrected T1 map was calculated using a Bloch-simulation look-up table generated using the acquisition parameters, with B1 correction based on the 3DREAM measured B1 map. The simulations included a range of T1 values between 1 and 3000 ms and a B1 range of 1% to 200% of the target flip angle.

## Data Records

WAND data^22^ are available for download from GIN (https://gin.g-node.org/CUBRIC/WAND).

### BIDS metadata and data organisation

All imaging data are organised using the Brain Imaging Data Structure (BIDS^32^) or, where no official BIDS recommendation exists, data are structured according to recommendations from BIDS extension proposals current at the time of acquisition. Details of data structure and decision-making related to non-official BIDS formats are included in the README file of the GitLab repository (https://git.cardiff.ac.uk/cubric/wand).

Data are organised first by subject, then session, then data type (e.g., “meg”, “func”, “dwi”, etc.). Sessions are organised such that a single session contains imaging data from only one device on one occasion (see Table S2 for details).

MRI data are stored in NifTI (Neuroimaging Informatics Technology Initiative) format with accompanying metadata stored in JSON format. MRS data are stored as Siemens TWIX files, using a BIDS-like structure but will not be “BIDS-valid”. MEG data are stored in .ds format according to CTF’s data organisation standards. Continuous recordings from physiological monitoring are stored as tab-separated values (TSV) files with accompanying metadata stored in JSON format. Timing and other properties of events recorded during functional MRI tasks are stored as TSV files with accompanying metadata in JSON format. Cognitive and questionnaire data are stored in TSV format with accompanying metadata/data dictionaries in JSON format.

## Technical Validation

BIDS validation was carried out using BIDS^32^ Validator (https://github.com/bids-standard/bids-validator). The dataset has been made as “valid” as possible, given the limitations of the BIDS framework at the time of publication.

For each session and each imaging modality, data were checked for motion and artefacts (see details of quality reports in *pre/post processing of data*, above). No data were excluded from the database due to image corruption.

The test-retest repeatability of microstructural sequences used in WAND has been reported previously^36^.

## Usage Notes

Download instructions, code and protocols are accessible through our GitLab repository: https://git.cardiff.ac.uk/cubric/wand. Users can access and download the whole dataset or can interact with, retrieve and link data (and metadata) using GIN (https://gin.g-node.org/), allowing for improved storage management and working practices. Detailed instructions for GIN download and WAND dataset access are provided in the git documentation.

## Supplementary information

Supplementary information

## Data Availability

Most imaging data are shared in their unprocessed form. Where pre-processing was necessary for sharing, code is available in the code folder of the git repository (https://git.cardiff.ac.uk/cubric/wand/-/tree/main/code). Preprocessing scripts required to summarise the S/LICI outcomes from TMS data are also provided. By making these data publicly available, we intend to bolster research into neuroimaging and related fields, contribute to the democratisation of MRI, and facilitate collaboration in MRI and MEG research.
