# Correction: WAND: A multi-modal dataset integrating advanced MRI, MEG, and TMS for multi-scale brain analysis

**DOI:** 10.1038/s41597-025-05476-w

**Published:** 2025-07-03

**Authors:** Carolyn B. McNabb, Ian D. Driver, Vanessa Hyde, Garin Hughes, Hannah L. Chandler, Hannah Thomas, Christopher Allen, Eirini Messaritaki, Carl J. Hodgetts, Craig Hedge, Maria Engel, Sophie F. Standen, Emma L. Morgan, Elena Stylianopoulou, Svetla Manolova, Lucie Reed, Matthew Ploszajski, Mark Drakesmith, Michael Germuska, Alexander D. Shaw, Lars Mueller, Holly Rossiter, Christopher W. Davies-Jenkins, Tom Lancaster, C. John Evans, David Owen, Gavin Perry, Slawomir Kusmia, Emily Lambe, Adam M. Partridge, Allison Cooper, Peter Hobden, Hanzhang Lu, Kim S. Graham, Andrew D. Lawrence, Richard G. Wise, James T. R. Walters, Petroc Sumner, Krish D. Singh, Derek K. Jones

**Affiliations:** 1 Cardiff University Brain Research Imaging Centre, School of Psychology, Cardiff University, Cardiff, UK; 2 Department of Psychology, Durham University, Durham, UK; 3 Department of Psychology, Royal Holloway, University of London, Egham, UK; 4 School of Psychology, Aston University, Birmingham, UK; 5 Department of Radiology, University of California Davis Medical Center, Sacramento, California USA; 6 Washington Singer Laboratories, University of Exeter, Exeter, UK; 7 Leeds Institute of Cardiovascular and Metabolic Medicine, University of Leeds, Leeds, UK; 8 The Russell H. Morgan Department of Radiology and Radiological Science, Johns Hopkins University School of Medicine, Baltimore, Maryland USA; 9 F.M. Kirby Research Center for Functional Brain Imaging, Kennedy Kreiger Institute, Baltimore, Maryland USA; 10 Department of Psychology, University of Bath, Bath, UK; 11 IBM Polska Sp. z o. o., Department of Content Design, Cracow, Poland; 12 University of Sheffield, Research Services, New Spring House, 231 Glossop Road, Sheffield, S10 2GW UK; 13 School of Philosophy, Psychology and Language Sciences, Dugald Stewart Building, University of Edinburgh, 3 Charles Street, Edinburgh, EH8 9AD UK; 14 Department of Neurosciences, Imaging, and Clinical Sciences, ‘G. D’Annunzio’ University of Chieti-Pescara, Chieti, Italy; 15 Institute for Advanced Biomedical Technologies, ‘G. D’Annunzio’ University of Chieti-Pescara, Chieti, Italy; 16 School of Medicine, Centre for Neuropsychiatric Genetics and Genomics, Cardiff University, Cardiff, UK

**Keywords:** Neuroscience, Human behaviour, Cognitive neuroscience

Correction to: *Scientific Data* 10.1038/s41597-024-04154-7, published online 06 February 2025

In the sentence beginning ‘For each trial, a white fixation dot...’ in this article, the text ‘3 cycles per degree’ should have read ‘1.5 cycles per degree’.

