# Supplementary material for: WAND: A multi-modal dataset integrating advanced MRI, MEG, and TMS for multi-scale brain analysis
Source: Sci Data. 2025 Feb 6;12:220. doi: 10.1038/s41597-024-04154-7 (PMC11803114; doi:10.1038/s41597-024-04154-7)
Supplement: Supplementary file 1 — Supplementary information [file 41597_2024_4154_MOESM1_ESM.pdf]

SUPPLEMENTARY MATERIALS

WAND: A multi-modal dataset integrating advanced MRI, MEG, and TMS for multi-scale brain analysis

Carolyn B McNabb[1], Ian D Driver[1], Vanessa Hyde[1], Garin Hughes[1], Hannah L Chandler[1], Hannah Thomas[1], Christopher Allen[2], Eirini Messaritaki[1], Carl J Hodgetts[1,3], Craig Hedge[4], Maria Engel[1], Sophie F Standen[1], Emma L Morgan[1], Elena Stylianopoulou[1], Svetla Manolova[1], Lucie Reed[1], Mark Drakesmith[1], Michael Germuska[5], Alexander D Shaw[6], Lars Mueller[7], Holly Rossiter[1], Christopher W Davies-Jenkins[8,9], Tom Lancaster[10], C John Evans[1], David Owen[1], Gavin Perry[1], Slawomir Kusmia[1,11], Emily Lambe[1], Adam M Partridge[1,12], Allison Cooper[1], Peter Hobden[1], Hanzhang Lu[8,9], Kim S Graham[1,13], Andrew D Lawrence[1,13], Richard G Wise[1,14.15], James T R Walters[16], Petroc Sumner[1], Krish D Singh[1], and Derek K Jones[1]

[1]Cardiff University Brain Research Imaging Centre, School of Psychology, Cardiff University, Cardiff, United Kingdom,
[2]Department of Psychology, Durham University, Durham, United Kingdom,
[3]Department of Psychology, Royal Holloway, University of London, Egham, United Kingdom,
[4]School of Psychology, Aston University, Birmingham, United Kingdom,
[5] Department of Radiology, University of California Davis Medical Center, Sacramento, California, USA,
[6]Washington Singer Laboratories, University of Exeter, Exeter, United Kingdom,
[7]Leeds Institute of Cardiovascular and Metabolic Medicine, University of Leeds, Leeds, United Kingdom,
[8]The Russell H. Morgan Department of Radiology and Radiological Science, Johns Hopkins University School of Medicine, Baltimore, Maryland, USA,
[9]F.M. Kirby Research Center for Functional Brain Imaging, Kennedy Kreiger Institute, Baltimore, Maryland, USA,
[10]Department of Psychology, University of Bath, Bath, United Kingdom,
[11]IBM Polska Sp. z o. o., Department of Content Design, Cracow, Poland
[12]University of Sheffield, Research Services, New Spring House, 231 Glossop Road, Sheffield, S10 2GW, UK
[13]School of Philosophy, Psychology and Language Sciences, Dugald Stewart Building, University of Edinburgh, 3 Charles Street, Edinburgh, EH8 9AD, UK
[14]Department of Neurosciences, Imaging, and Clinical Sciences, 'G. D'Annunzio' University of Chieti-Pescara, Chieti, Italy,
[15]Institute for Advanced Biomedical Technologies, 'G. D'Annunzio' University of Chieti-Pescara, Chieti, Italy
[16]School of Medicine, Centre for Neuropsychiatric Genetics and Genomics, Cardiff University, Cardiff, United Kingdom

TABLE OF CONTENTS

Table S1. Reasons for exclusion from the Welsh Advanced Neuroimaging Database 3
Table S2. Imaging data acquired as part of the Welsh Advanced Neuroimaging Database. Session numbers and data type are provided to assist researchers in locating the data in the public repository and have been organised using the Brain Imaging Data Structure (BIDS). Abbreviations: MPRAGE - Magnetization Prepared Rapid Gradient Echo; BOLD – blood oxygen level dependent; EPI – echo planar imaging; pCASL – Pseudo-continuous arterial spin labelling; semi-LASER - semi-localization by adiabatic selective refocusing; MEGA-PRESS - Meshcher-Garwood Point RESolved Spectroscopy; TRUST - T2 relaxation under spin tagging... 5
Table S3. Summary of MRI parameters for each acquisition sequence. Abbreviations: TR – repetition time; TE – echo time; FOV – field of view; TI – inversion time; A>>P – anterior to posterior; MPRAGE - Magnetization Prepared Rapid Gradient Echo; BOLD – blood oxygen level dependent; EPI – echo planar imaging; SPGR - spoiled gradient-recalled echo sequence; SPGR-IR - inversion recovery-prepped spoiled gradient-recalled echo sequence; SSFP - steady-state free precession; pCASL – Pseudo-continuous arterial spin labelling; semi-LASER - semi-localization by adiabatic selective refocusing; MEGA-PRESS - Meshcher-Garwood Point RESolved Spectroscopy; TRUST - T2 relaxation under spin tagging; MP2RAGE - magnetization prepared 2 rapid gradient echoes. 7
Table S4. Cognitive tasks and questionnaires included in the Welsh Advanced Neuroimaging Database. Abbreviations: UPPS-P - Urgency-Premeditation-Perseverance-Sensation Seeking-Positive Urgency 12
Table S5. Summary statistics for questionnaire data included in the Welsh Advanced Neuroimaging Database. 13
Table S6. Summary statistics for cognitive tasks included in the Welsh Advanced Neuroimaging Database. 16

Table S1. Reasons for exclusion from the Welsh Advanced Neuroimaging Database

Reason for exclusion	Number of people excluded
depression	71
time	51
not MRI compatible	49
did not specify	48
medication	45
migraines	32
tattoo location	32
psychiatric diagnosis	23
concussion	22
location	21
anxiety disorder	16
diabetes	11
claustrophobia	10
copper IUD	8
pregnancy/breastfeeding	7
age	6
neurological - did not specify	6
regular smoker	6
ADHD	5
pain killers	5
stroke	5
rheumatoid arthritis	4
PTSD	3
titanium plates	3
bipolar	2
brain tumour	2
drug use	2
haemorrhage	2
HIV	2
Interest	2
metal in body	2
multiple sclerosis	2
traumatic brain injury	2
ADHD and Anxiety Disorder	1
aneurysm	1
anorexia	1
Asthma	1
autism	1
cancer	1
carpal tunnel syndrome	1

charcot marie tooth disease	1
chiara I malformation	1
epilepsy	1
family history	1
hernia mesh	1
high blood pressure	1
hydrocephalus	1
illness	1
long-term illness	1
narcolepsy	1
other	1
pacemaker	1
panic disorder	1
parkinson's disease	1
restless leg syndrome	1
sterilisation	1
tourettes	1

Table S2. Imaging data acquired as part of the Welsh Advanced Neuroimaging Database. Session numbers and data type are provided to assist researchers in locating the data in the public repository and have been organised using the Brain Imaging Data Structure (BIDS). Abbreviations: MPRAGE - Magnetization Prepared Rapid Gradient Echo; BOLD – blood oxygen level dependent; EPI – echo planar imaging; pCASL – Pseudo-continuous arterial spin labelling; semi-LASER - semi-localization by adiabatic selective refocusing; MEGA-PRESS - Meshcher-Garwood Point RESolved Spectroscopy; TRUST - T2 relaxation under spin tagging.

Session	Data type	Acquisition sequence	Functional task	File naming pattern
**Magnetoencephalography (MEG; ses-01)**	meg		Rest	*task-resting
	meg		Visual Gamma	*task-visual
	meg		Simon Task	*task-simon
	meg		40Hz auditory stimulus / finger abduction task	*task-auditorymotor
	meg		Mismatch negativity	*task-mmn
**Diffusion and quantitative 3T MRI (300 mT/m; ses-02)**	anat	T1-weighted structural image (MPRAGE)		*T1w
	dwi	Multi-shell high angular resolution diffusion imaging		*acq-CHARMED_dir*_dwi
	dwi	Diffusion-weighted imaging with variable diffusion time		*acq-AxCaliber_dir*_dwi
	anat	Quantitative magnetisation transfer imaging		*QMT
	anat	Spoiled and unspoiled gradient echoes		*VFA
**3T functional and perfusion MRI (ses-03)**	anat	T1-weighted structural image (MPRAGE)		*T1w
	anat	T2-weighted turbo spin echo		*rec-nlgradcorr_T2w
	func	Blood oxygen level dependent echo planar imaging (BOLD EPI)	Category localiser task	*task-categorylocaliser_run*_bold
	func	BOLD EPI	Reversal learning task	*task-reversallearning_bold
	func	BOLD EPI	Rest	*task-rest_bold
	perf	Pseudo-continuous arterial spin labelling (pCASL)		*acq-PCASL_cbf
	perf	T2-relaxation-under-spin-tagging (TRUST) – venous blood oxygenation		*acq-TRUST_cbf
	perf	T1 inversion recovery – haemoglobin		*acq-InvRec_cbf
**7T Magnetic resonance spectroscopy (MRS; ses-04)**	anat	T1-weighted structural image (MPRAGE)		*T1w
	mrs	semi-LASER – left sensorimotor (metabolite and water reference)		*acq-slaser_voi-smleft*
	mrs	semi-LASER – anterior cingulate (metabolite and water reference)		*acq-slaser_voi-anteriorcingulate*
	mrs	semi-LASER – occipital (metabolite and water reference)		*acq-slaser_voi-occipital*
	mrs	semi-LASER – right auditory (metabolite and water reference)		*acq-slaser_voi-rightauditory*
	anat	T1-weighted structural image (MPRAGE)		*T1w

**3T Magnetic resonance spectroscopy (MRS; ses-05)**	mrs	MEGA-PRESS – left sensorimotor (metabolite and water reference)		*acq-mega_voi-smleft*
	mrs	MEGA-PRESS – anterior cingulate (metabolite and water reference)		*acq-mega_voi-anteriorcingulate*
	mrs	MEGA-PRESS – occipital (metabolite and water reference)		*acq-mega_voi-occipital*
	mrs	MEGA-PRESS – right auditory (metabolite and water reference)		*acq-mega_voi-rightauditory*
**7T structural/functional MRI (ses-06)**	anat	T1-weighted structural image (MP2RAGE)		*MP2RAGE
	anat	T2-weighted turbo spin echo (slices orthogonal to hippocampal long axis)		*T2w
	func	BOLD EPI	Rest	*task-rest_bold
	anat	Multi-echo gradient echo (mGRE)		*MEGRE
**3T metabolic MRI (ses-07)**	perf	TRUST – venous blood oxygenation		*TRUST_cbf
	perf	T1 inversion recovery – haemoglobin		*acq-InvRec_cbf
	perf	Dual-excitation pCASL	Breath-hold	*acq-PCASL-task-breathhold_cbf
	anat	Single-echo gradient echo (GRE)		*T2starw
	func	Spin echo EPI	Breath-hold	*task-breathhold_bold
	perf	Dual-excitation pCASL	Rest	*acq-PCASL-task-rest_cbf
	perf	Dual-excitation pCASL	Breath-hold + visual stimulation (checkerboard)	*acq-PCASL-task-breathholdchecker_cbf
**Transcranial magnetic stimulation (ses-08)**	tms		Short and Long Interval Intra Cortical Inhibition	*SICIdatam

Table S3. Summary of MRI parameters for each acquisition sequence. Abbreviations: TR – repetition time; TE – echo time; FOV – field of view; TI – inversion time; A>>P – anterior to posterior; MPRAGE - Magnetization Prepared Rapid Gradient Echo; BOLD – blood oxygen level dependent; EPI – echo planar imaging; SPGR - spoiled gradient-recalled echo sequence; SPGR-IR - inversion recovery-prepped spoiled gradient-recalled echo sequence; SSFP - steady-state free precession; pCASL – Pseudo-continuous arterial spin labelling; semi-LASER - semi-localization by adiabatic selective refocusing; MEGA-PRESS - Meshcher-Garwood Point RESolved Spectroscopy; TRUST - T2 relaxation under spin tagging; MP2RAGE - magnetization prepared 2 rapid gradient echoes.

Session	Acquisition	Parameters
**Diffusion and quantitative 3T MRI (300 mT/m; ses-02)**	T1-weighted anatomical	TR: 2300 ms<br>TE: 2 ms<br>Flip Angle: 9°<br>FOV: 256 x 256 x 192 mm^3<br>Voxel Size: 1 x 1 x 1 mm^3<br>TI: 857 ms<br>Phase-encoding: A>>P
	Multi-shell diffusion-weighted MRI	TR: 3000 ms<br>TE: 59 ms<br>FOV: 220 x 220 x 132 mm^3<br>Voxel Size: 2 x 2 x 2 mm^3<br>Phase-encoding: A>>P and P>>A<br>Diffusion gradient: δ = 7 ms, Δ = 24 ms<br>b-values: b=200, 500, 1200, 2400, 4000, 6000 s/mm^2)
	Diffusion-weighted imaging with variable diffusion time	TR: 3900 ms<br>TE: 80 ms<br>FOV: 220 x 220 x 132 mm^3<br>Voxel Size: 2 x 2 x 2 mm^3<br>Phase-encoding: A>>P and P>>A<br>Diffusion gradient: δ = 7 ms<br>Δ and b-values: Multiple (Δ = 18, 30, 42, 55 ms with various b-value pairs up to b = 7750 and 15500 s/mm^2)
	Spoiled and unspoiled gradient echo	SPGR: TR 4 ms, TE 1.9 ms, 8 flip angles (3 - 18°)<br>SPGRIR: TR 4 ms, TE 1.9 ms, flip angle 5°<br>SSFP: TR 4.54 ms, TE 2.27 ms, 8 flip angles (10 - 60°)<br>FOV: 220 × 220 × 179 mm^3<br>Voxel Size: 1.72 x 1.72 x 1.72 mm^3<br>Phase-encoding: A>>P
	Quantitative magnetization transfer (qMT)	TR: 55 ms<br>TE: 2.1 ms<br>FOV: 220 x 220 x 179 mm^3<br>Voxel Size: 1.72 x 1.72 x 1.72 mm^3<br>MT-weighted images: 11 combinations of flip angle/offset<br>Phase-encoding: A>>P<br>Turbo factor: 4
**3T functional and perfusion MRI (ses-03)**	T1-weighted anatomical	TR: 2250 ms<br>TE: 3.06 ms<br>Flip Angle: 9°

		FOV: 256 x 288 x 176 mm^3<br>Voxel Size: 1 x 1 x 1 mm^3<br>TI: 850 ms<br>Phase-encoding: A>>P
	T2-weighted anatomical	TR: 8000 ms<br>TE: 82 ms<br>Echo train length: 17<br>Echo spacing: 8.22 ms<br>Flip Angle: 120°<br>FOV: 256 x 256 mm^2<br>Voxel Size: 1 x 1 mm^2<br>Slices: 88 (2 mm thick, contiguous)
	BOLD fMRI (task-based and resting-state)	TR: 2000 ms<br>TE: 30 ms<br>Flip Angle: 70°<br>FOV: 192 x 192 x 160 mm^3<br>Voxel Size: 2.0 x 2.0 x 2.0 mm^3<br>Phase-encoding: A>>P<br>Multiband Acceleration Factor: 4
	Pseudocontinuous arterial spin labelling (pCASL)	TR: 4600 ms<br>TE: 11 ms<br>Post label delay: 2000 ms<br>Tag duration: 1800 ms<br>FOV: 320 x 320 mm^2<br>Voxel Size: 3.4 x 3.4 x 5.0 mm^3<br>Slices: 22 (5 mm thick)
	T2 Relaxation under Spin Tagging (TRUST)	TR: 3000 ms<br>TE: 3.9 ms<br>Effective echo times: 0, 40, 80, 160 ms<br>FOV: 220 x 220 mm^2<br>Voxel Size: 3.4 x 3.4 x 5.0 mm^3
	T1 Inversion Recovery	TR and ΔTR: 150 ms<br>TE: 22 ms<br>Post label delay: 1800 ms<br>Flip Angle: 90°<br>FOV: 240 x 240 mm^2<br>Voxel Size: 3.4 x 3.4 x 5.0 mm^3<br>Slices: 1 (3 mm thick)
	Gradient Echo Field Maps	TR: 434 ms<br>TE1: 4.92 ms<br>TE2: 7.38 ms<br>Flip Angle: 60°<br>FOV: 96 x 96 mm^2<br>Voxel Size: 2.0 x 2.0 x 6.0 mm^3
**7T Magnetic resonance spectroscopy (MRS; ses-04)**	B1 Inhomogeneity Map	TR: 5000 ms<br>TE1: 0.9 ms<br>TE2: 1.55 ms<br>Flip Angle1: 60°<br>FOV: 200 x 200 x 200 mm^3<br>Voxel Size: 4.5 x 4.5 x 4.5 mm^3

		Phase encoding direction: R>>L
	T1-weighted anatomical	TR: 2200 ms<br>TE: 3.02 ms<br>Flip Angle: 7°<br>FOV: 224 x 224 x 157 mm^3<br>Voxel Size: 0.7 x 0.7 x 0.7 mm^3<br>TI: 1050 ms
	Metabolite and water reference data (semi-LASER)	TR: 5230 ms<br>TE: 78 ms<br>Voxel Size: Various VOIs (30 x 30 x 30 mm^3, 25 x 30 x 40 mm^3)<br>Frequency offset: 1.7 ppm for metabolites, 0 ppm for water<br>Spectral width: 3000 Hz
**3T Magnetic resonance spectroscopy (MRS; ses-05)**	T1-weighted anatomical	TR: 2250 ms<br>TE: 3.06 ms<br>Flip Angle: 9°<br>FOV: 256 x 288 x 176 mm^3<br>Voxel Size: 1 x 1 x 1 mm^3<br>TI: 850 ms
	GABA-edited MRS and water reference data (MEGA-PRESS)	TR: 2000 ms<br>TE: 68 ms<br>Voxel Size: Various VOIs (30 x 30 x 30 mm^3, 25 x 30 x 40 mm^3)<br>Frequency offset: 1.7 ppm for metabolites, 0 ppm for water<br>Spectral width: 2000 Hz
**7T structural/functional MRI (ses-06)**	B1 Inhomogeneity Map	TR: 5000 ms<br>TE1: 0.9 ms<br>TE2: 1.55 ms<br>Flip Angles: 60°, 8°, 3°<br>FOV: 200 x 200 x 200 mm^3<br>Voxel Size: 4.5 x 4.5 x 4.5 mm^3<br>Phase-encoding: R>>L
	T1-weighted anatomical (MP2RAGE)	TR: 3500 ms<br>TE: 2.64 ms<br>Flip Angles: 5° (1st), 2° (2nd)<br>FOV: 224 x 224 x 157 mm^3<br>Voxel Size: 0.7 x 0.7 x 0.7 mm^3<br>TI1: 725 ms<br>TI2: 2150 ms<br>Phase-encoding: A>>P
	T2-weighted anatomical	TR: 13000 ms<br>TE: 75 ms<br>Echo Spacing: 15.1 ms<br>Flip Angle: 120°<br>Echo Train Length: 9<br>FOV: 220 x 220 mm^2<br>Voxel Size: 0.4 x 0.4 mm^2<br>In-plane Interpolation: 0.2 x 0.2 mm^2

	Resting-state BOLD fMRI	TR: 1500 ms<br>TE: 25 ms<br>Flip Angle: 65°<br>Echo Spacing: 0.72 ms<br>FOV: 192 x 192 mm^2<br>Voxel Size: 1.5 x 1.5 x 1.5 mm^3<br>Multiband Acceleration factor: 4<br>Phase Encoding: A>>P
	Spin Echo EPI for Distortion Correction	TR: 2000 ms<br>TE: 45 ms<br>Phase Encoding: A>>P and P>>A
	Multi-echo 3D Gradient Echo	TR: 39 ms<br>Flip Angle: 11°<br>Echo Times: 7 echoes, 5 to 35 ms (5 ms increments)<br>FOV: 224 x 224 x 224 mm^3<br>Voxel Size: 0.67 x 0.67 x 0.67 mm^3<br>Slices: 192<br>Bandwidth: 290 Hz/px<br>Undersampling: 2x2
**3T metabolic MRI (ses-07)**	TRUST (T2 Relaxation under Spin Tagging)	TR: 3000 ms<br>TE: 3.9 ms<br>Echo Times: 0, 40, 80, 160 ms<br>FOV: 220 x 220 mm^2<br>Voxel Size: 3.4 x 3.4 x 5 mm^3<br>TI: 1020 ms<br>Phase Partial Fourier: 6/8
	T1 Inversion Recovery	TR: 150 ms<br>TE: 22 ms<br>Post Label Delay: 1800 ms<br>Flip Angle: 90°<br>FOV: 240 x 240 mm^2<br>Slice Thickness: 3 mm
	3D Phase Contrast	TR: 40.65 ms<br>TE: 5.6 ms<br>Flip Angle: 10°<br>FOV: 180 x 240 x 78 mm^3<br>Voxel Size: 0.47 x 0.47 x 1.30 mm^3<br>Venc: 75 cm/s
	Dual excitation (DEXI) pCASL	TR: 4.4 s<br>TE1: 10 ms<br>TE2: 30 ms<br>FOV: 3.4 x 3.4 mm^2<br>Slice Thickness: 6 mm (33% gap)<br>Task: Breath-hold and resting-state
	M0 scans for CBF quantification	TR: 6000 ms<br>TE: 10 ms<br>Flip Angle: 90°<br>Phase Encoding: A>>P and P>>A (matched with DEXI dataset)

Spin-echo EPI	TR: 2500 ms<br>TE: 90 ms<br>Echo Spacing: 0.55 ms<br>Voxel Size: 3.4 x 3.4 x 6.5 mm^3<br>Slices: 15<br>Task: Breath-hold
Single-echo 3D Gradient Echo	TR: 20 ms<br>TE: 14 ms<br>Flip Angle: 15°<br>Voxel Size: 0.8 x 0.8 x 1.0 mm^3<br>FOV: 230 x 230 x 144 mm^3<br>Slices: 144<br>Bandwidth: 220 Hz/px

Table S4. Cognitive tasks and questionnaires included in the Welsh Advanced Neuroimaging Database. Abbreviations: UPPS-P - Urgency-Premeditation-Perseverance-Sensation Seeking-Positive Urgency

**Cognitive tasks**	**Number of Participants**	**Name of file**
Choice reaction-time tasks	174	TwoChoice.tsv; FourChoice.tsv
Logical memory task	178	cog_tasks.tsv
Vocabulary test	178	cog_tasks.tsv
Matrix reasoning (WASI-II)	178	cog_tasks.tsv
Verbal fluency test	178	cog_tasks.tsv
Balloon analogue risk task	173	BART.tsv
**Participant demographics**		
Demographic interview	178	participants.tsv
**Neuropsychiatric interview**		
Mini International Neuropsychiatric Interview	178	MINI.tsv
**Trait questionnaires**		
Short UPPS-P Impulsive Behaviour Scale	160	questionnaires.tsv; WAND_SUBSCALE_SCORES.tsv
Generalized Anxiety Disorder 7-item scale	160	questionnaires.tsv; WAND_SUBSCALE_SCORES.tsv
Anxiety Depression Distress Inventory	160	questionnaires.tsv; WAND_SUBSCALE_SCORES.tsv
Global Physical Activity Questionnaire	160	questionnaires.tsv; WAND_SUBSCALE_SCORES.tsv
Emotion Regulation Questionnaire	160	questionnaires.tsv; WAND_SUBSCALE_SCORES.tsv
Big Five Inventory-2 short form	160	questionnaires.tsv; WAND_SUBSCALE_SCORES.tsv
Schizotypal Personality Questionnaire	160	questionnaires.tsv; WAND_SUBSCALE_SCORES.tsv
Single Item Need to Belong Scale	160	questionnaires.tsv; WAND_SUBSCALE_SCORES.tsv
Survey of Autobiographical Memory	160	questionnaires.tsv; WAND_SUBSCALE_SCORES.tsv
Navigational Strategies Questionnaire	160	questionnaires.tsv; WAND_SUBSCALE_SCORES.tsv
Mind Wandering Questionnaire	160	questionnaires.tsv; WAND_SUBSCALE_SCORES.tsv
Fazio Laterality Inventory	160	questionnaires.tsv; WAND_SUBSCALE_SCORES.tsv
Hospital Anxiety and Depression Scale	160	questionnaires.tsv; WAND_SUBSCALE_SCORES.tsv
Visual Vertigo analogue Scale	160	questionnaires.tsv; WAND_SUBSCALE_SCORES.tsv
Adult Autism Spectrum Quotient – short version	160	questionnaires.tsv; WAND_SUBSCALE_SCORES.tsv
Visual discomfort images	160	questionnaires.tsv; WAND_SUBSCALE_SCORES.tsv
Adolescent/Adult Sensory Profile	160	questionnaires.tsv; WAND_SUBSCALE_SCORES.tsv
Migraine Screening Questionnaire	160	questionnaires.tsv; WAND_SUBSCALE_SCORES.tsv

Table S5. Summary statistics for questionnaire data included in the Welsh Advanced Neuroimaging Database.

Trait questionnaire	Subscale	Mean	Std	Min	Max	Cronbach's Alpha
**Anxiety Depression Distress Inventory**	General distress	15.69	6.23	9	39	0.92
	Positive affect	27.98	6.55	11	45	0.89
	Somatic anxiety	11.2	3.02	9	24	0.75
**Adult Autism Spectrum Quotient**	Social skills	13.59	4.44	7	28	0.85
	Routine	8.84	2.26	4	14	0.58
	Switching	9.07	2.26	4	16	0.61
	Imagination	15.77	3.59	8	26	0.67
	Numbers and patterns	10.74	3.13	5	20	0.69
**Adolescent/Adult Sensory Profile**	Low registration	30.81	6.69	17	48	0.75
	Sensation seeking	49.29	6.26	33	67	0.58
	Sensory sensitivity	34.67	7.64	20	58	0.72
	Sensory avoiding	33.8	7.82	19	60	0.77
**Big Five Inventory**	Extraversion	3.37	0.8	1.17	5	0.76
	Agreeableness	4.05	0.72	1.33	5	0.78
	Conscientiousness	3.71	0.73	2	5	0.74
	Negative emotionality	2.37	0.91	1	4.83	0.85
	Openness	3.79	0.71	1.67	5	0.71
**Emotion Regulation Questionnaire**	Reappraisal	4.99	1.08	2.17	7	0.87
	Suppression	3.58	1.26	1	6.75	0.75
**Fazio Laterality Inventory**	Total	79.59	17.27	2	100	0.91
**Generalized Anxiety Disorder 7-item scale**	Total	2.94	3.02	0	16	0.81
**Global Physical Activity Questionnaire**	Total metabolic equivalents	4123.42	3964.66	0	20880	-
**Hospital Anxiety and Depression Scale**	Anxiety	5.22	3.66	0	15	0.82
	Depression	2.67	2.72	0	15	0.75

**Migraine Screening Questionnaire**	Total	0.82	0.98	0	5	0.52
**Mind Wandering Questionnaire**	Total	3.61	0.91	1.4	6	0.82
**Navigational Strategies Questionnaire**	Map total	4.96	2.73	0	12	-
	Non map total	5.24	2.5	0	12	-
	Map preference	-0.28	4.96	-11	12	-
**Survey of Autobiographical Memory**	Episodic	3.3	0.76	1.12	5	0.82
	Factual	3.28	0.75	1.33	5	0.7
	Spatial	3.68	0.75	1.67	5	0.75
	Future	3.78	0.68	1	5	0.78
**Single Item Need to Belong Scale**	Total	3.38	1.18	1	5	-
**Schizotypal Personality Questionnaire**	Total	12.43	10.39	0	53	0.93
	Ideas reference	1.15	1.78	0	8	0.78
	Social anxiety	2.61	2.43	0	8	0.83
	Odd beliefs	0.3	0.81	0	6	0.67
	Perceptual	0.74	1.34	0	9	0.71
	Odd behaviour	1.09	1.73	0	6	0.83
	No friends	1.82	2.18	0	9	0.81
	Odd speech	2.1	2.05	0	9	0.74
	Affect	1.39	1.7	0	8	0.74
	Suspiciousness	1.23	1.72	0	8	0.77
**Short UPPS-P Impulsive Behaviour Scale**	Negative urgency	2.02	0.62	1	4	0.73
	Lack of perseverance	1.76	0.44	1	3.25	0.66
	Lack of premeditation	1.74	0.47	1	3	0.77
	Sensation seeking	2.76	0.67	1.25	4	0.66
	Positive Urgency	1.64	0.51	1	3.75	0.75
**Visual discomfort images**	Low	0.76	0.95	0	5.9	0.81
	High	1.72	1.76	0	8	0.93

**Visual Vertigo analogue Scale**	Severity	6.11	8.98	0	47.78	0.82

Note: N=160 except for the GPAQ, where N=146. Some participants gave ambiguous responses to questions asking in HH:MM how much time was spent on physical activity per day (e.g. "7"). Data for participants which were judged to be ambiguous were not included in these statistics. We recommend that those interested in the GPAQ re-evaluate the raw data and inclusions/exclusions.

Table S6. Summary statistics for cognitive tasks included in the Welsh Advanced Neuroimaging Database.

Task	Subscale	Mean	Std	Min	Max	Split-half reliability
**Two choice RT**	Accuracy	94.7	7.2	10.5	99.8	0.98
	Mean RT	445	56	330	743	0.99
**Four choice RT**	Accuracy	94.6	3.4	83.3	100.0	0.89
	Mean RT	381	73	265	922	1.00
**BART**	Mean adjusted pumps	33.7	14.1	9.6	76.9	0.97
**Logical memory**	Story B immediate recall	15.0	4.0	2.0	23.0	-
	Story C immediate recall	12.3	3.5	4.0	22.0	-
	Story B delayed recall	12.7	4.4	0.0	22.0	-
	Story C delayed recall	11.1	3.7	1.0	22.0	-
	Story B delayed recognition	12.9	2.1	0.0	22.0	-
	Story C delayed recognition	12.3	2.0	0.0	15.0	-
**WASI II**	Vocabulary (raw)	39.8	5.1	27.0	52.0	-
	Matrix Reasoning (raw)	19.7	3.5	4.0	26.0	-
**Verbal fluency**	Phonemic (f words)	16.1	4.8	5.0	31.0	-
	Semantic (animals)	24.8	6.5	10.0	43.0	-
	Social (friends' names)	22.5	6.6	9.0	43.0	-
	Spatial (bedroom objects)	23.0	5.6	9.0	39.0	-

Note. Split-half reliability coefficients are calculated from odd and even trials with Spearman-Brown correction applied
